# Digestive α-L-fucosidase activity in *Rhodnius prolixus* after blood feeding: effect of secretagogue and nutritional stimuli

**DOI:** 10.3389/fphys.2023.1123414

**Published:** 2023-07-19

**Authors:** Maiara do Valle Faria Gama, Yasmim do Nascimento Alexandre, João Mario Pereira da Silva, Daniele Pereira Castro, Fernando Ariel Genta

**Affiliations:** ^1^ Instituto Oswaldo Cruz, Fundação Oswaldo Cruz, Rio de Janeiro, Brazil; ^2^ Instituto Nacional de Ciência e Tecnologia em Entomologia Molecular, Rio de Janeiro, Brazil

**Keywords:** α-fucosidase, *Rhodnius prolixus*, blood digestion, carbohydrase, vector physiology

## Abstract

**Introduction:** *Rhodnius prolixus* (Hemiptera: Reduviidae) is an important vector of *Trypanosoma cruzi*, the causative agent of Chagas Disease. This insect is a model for the study of insect physiology, especially concerning the digestion of blood. Among the enzymes produced in the midgut of *R. prolixus* after blood feeding there is a α-L-fucosidase activity. There are very few studies on α-L-fucosidase of insects, and the role of *R. prolixus* α-L-fucosidase is still not clear. In this work, we tested if the mechanism for production of this enzyme is similar to the observed for proteases, a secretatogue mechanism that respond to the protein contents of the meal.

**Methods:** We tested if specific proteins or sugars elicit this response, which may help to understand the nature of the physiological substrate for this enzyme.

**Results:** In general, our results showed that the Anterior Midgut was the only midgut fraction that responds to the blood meal in terms of α-L-fucosidase production. Besides that, this response was not triggered by midgut distension or by ingestion of the blood cell fraction. Conversely, the enzyme was produced after feeding with the plasma fraction. However, the production of α-L-fucosidase was also triggered by different biochemical stimuli, as protein or fucoidan ingestion.

**Discussion:** This suggested that the production of the enzyme in the anterior midgut was a general physiological response under control of different convergent signals. Besides that, the comparison between different treatments for artificial blood feeding showed that heparinated blood was the choice with minor side effects for the study of the midgut α-L-fucosidase, when compared to defibrinated or citrated blood.

## 1 Introduction

*Rhodnius prolixus* belongs to the *Reduviidae* family, *Triatominae* subfamily (Schofield and Galvão, 2009), being the main vector of Chagas disease in Colombia, Venezuela, and Central America^1^ (Coura, 2015). *R. prolixus* is a hematophagous hemipteran that has been widely used as a study model in insect physiology due to its easy maintenance in the laboratory (Wigglesworth, 1933; Garcia et al., 1975; Azambuja et al., 2017). Several studies have focused on the machinery involved in blood digestion in this insect (revised in Garcia et al., 2010; Gutierrez-Cabrera et al., 2016; Oliveira and Genta, 2021). The recent description of several transcriptomes (Ribeiro et al., 2014) and the full genome sequence of *R. prolixus* (Mesquita et al., 2015) reinforce its status as a model species for studies on triatomine physiology. As other hemipterans, *R. prolixus* lacks a midgut chitinous peritrophic membrane, secreting instead a lipoproteic perimicrovillar membrane. The gut of *R. prolixus* is divided in 4 main parts, the foregut, the anterior midgut, the posterior midgut and the hindgut. The foregut includes the pharynx and esophagus, and its responsible for the blood intake. The anterior midgut stores the blood meal and is the place where the initial hemolysis and water absorption occur. The posterior midgut is the main site for hemoglobin breakdown, where major amounts of proteases are secreted, and the hindgut is the compartment where feces and urine are secreted and stored until the next meal (Oliveira and Genta, 2021). Differently from the observed in other blood sucking vectors as mosquitoes, the midgut pH in triatomines is slightly acidic (around 5.5), and most digestive enzymes have optimum pHs from strong acidic (e.g., 2 for cathepsin D) to slight acidic (e.g., 6.5 for amylase) (Terra et al., 1988).

Historically, studies on *R. prolixus* gut biochemistry have focused mainly on the digestion of blood proteins, with the description and characterization of a diverse array of cathepsin-like proteases, such as cathepsin B, L and D, that are mainly secreted to the contents of the posterior midgut (Terra et al., 1988; Henriques et al., 2017; 2021; Pimentel et al., 2020; Oliveira and Genta, 2021). A relevant feature of the secretion of gut proteases in *R. prolixus* is the pattern of production of these enzymes, with an increase in activity of almost ten times in the days following a blood meal in the posterior midgut. It has been suggested that the secretion of these activities follow a secretagogue mechanism, i.e., promoted by a stimulating substance, because their production depends on the concentration of protein in the meal (Garcia and Garcia, 1977). However, more recent investigations of the expression patterns of protease genes and activities, with different and sensitive approaches as RT-PCR, fluorescent substrates, and proteomics, have shown that not only there are significant protease activities in the anterior midgut, but also several distinct midgut proteases being expressed at different time windows along the digestion of blood (Ouali et al., 2020; 2021; Henriques et al., 2021).

Less studies on *R. prolixus* gut physiology have focused on carbohydrate digestion. Despite their elusive role, due to the low carbohydrate contents of the blood, it has been shown that several glycosidases as α- and β-glucosidase, α- and β-mannosidase, α- and β-galactosidase, α- and β-N-acetyl-galactosaminidase, α-L-fucosidase, β-N-acetylglucosaminidase, and lysozyme are produced after blood feeding either in the anterior and posterior midgut (Ribeiro and Pereira, 1984)^2^. Besides that, activities of amylase, lysozyme, α-galactosidase, α-glucosidase, α-mannosidase, β-glucosidase, β-mannosidase, and β-N-acetylglucosaminidase were described and characterized to some detail (Terra et al., 1988). Interestingly, it has been proposed that these enzymes may act on the cell wall of the bacterial symbiont *Rhodococcus rhodni* (Ribeiro and Pereira, 1984)^3^. However, the amylase activity observed in the anterior midgut seems to be derived from the bacterial symbiont, not produced by the insect midgut cells (Terra et al., 1988).

Among the diverse digestive carbohydrases that were previously described in *R. prolixus*, there is a significant activity of a α-L-fucosidase (Ribeiro and Pereira, 1984; Profeta et al., 2017). α-L-fucosidase (EC 3.2.1.51) belongs typically to glycoside hydrolase family 29 (GH29) (Drula et al., 2022). This exoglycosidase hydrolyzes fucose from oligosaccharides and glycoconjugates (Shaikh et al., 2013). Besides that, L-fucose is a common component of many glycans found in most of the plasma glycoproteins, and in the mucopolysaccharides and mucolipids of various human and animal tissues (Durand et al., 1969).

Conversely, digestive α-L-fucosidases have been identified in molluscs (Reglero and Cabezas, 1976; Endo et al., 1993; Berteau et al., 2002; Perrella et al., 2015; Escobar-Correas et al., 2019), ticks (Moreti et al., 2013), spiders (Perrela et al., 2018b) and bacteria (Wu et al., 2021; Zhou et al., 2022). The knowledge on insect α-L-fucosidases is very scarce, and, despite its initial description and characterization, the role of *R. prolixus* midgut α-L-fucosidase during blood digestion has not been studied in detail. There is no clear indication of which would be the physiological substrate of this enzyme and, besides that, it is not clear if the production mechanism for this activity follows the same pattern that was observed for proteases, e.g., response to the protein content of the meal. In this work, we tested if ingestion of particular blood fractions or specific molecules such as proteins and sugars was able to stimulate the production of the midgut α-L-fucosidase, aiming to understand the physiological role of this enzyme and to clarify the mechanism of their production after blood feeding.

## 2 Materials and methods

### 2.1 Reagents

All reagents used were from Sigma-Aldrich^®^. Some specific reagents used were Trehalose (Product N⁰ T9532), Fucose (Product N⁰ F2252), Fucoidan (Product N⁰ F8190),bovine hemoglobin (Product N⁰ H2500), human hemoglobin (Product N⁰ H7379), rabbit albumin (Product N⁰ A0764), bovine albumin (Product N⁰ A9418), and 4-methylumbelliferyl α-L-fucopyranoside (Product N⁰ M8527).

### 2.2 Insects

*Rhodnius prolixus* (Hemiptera: Reduviidae) were obtained from the colony of Laboratório de Bioquímica e Fisiologia de Insetos (IOC/Fiocruz). Insects were maintained at 28°C, 70% relative humidity as described in Azambuja and Garcia (1997). For this work only male adults were used, to minimize the interference of copulatory and egg development status in the gut physiology. Besides that, using males the impact in the bug colony was reduced.

### 2.3 Blood fractions

Rabbit blood was aliquoted into 15 mL falcon tubes and centrifuged at 10°C for 10 min at 5,000 x g. The supernatant (plasma) was separated from the precipitate (cell fraction) and transferred into a new tube. The precipitate was resuspended in Phosphate Buffered Saline (PBS), pH 7.2, to the initial volume. Washed cell fraction was obtained by repeating the process of centrifuging and resuspending cells in PBS 3 times, discarding the supernatant (Garcia and Garcia, 1977).

### 2.4 Feeding with artificial diets

Insects were fed with defibrinated, citrated, or heparinized rabbit blood, and solutions of specific proteins or sugars using an artificial feeder (Azambuja and Garcia, 1997).

Trehalose, Fucose, and Fucoidan were prepared at 10% (w/v) in PBS while bovine hemoglobin (BH), human hemoglobin (HH), rabbit albumin (RA), and bovine albumin (BA) were prepared at a concentration of 13 mg/mL in PBS (Henriques et al., 2016).

The insects were individually weighed before and after feeding. In addition, the insects that did not engorge were counted and the mortality rate was measured at 5 days after feeding. These experiments and measurements were performed with groups of 20 insects in each treatment/condition.

### 2.5 Sample preparation

Experiments were performed with adult males, and samples consisted of pools of 5 insects or individual insects, depending on the experiment. For activity and protein assays, 5 days after blood feeding the insects were immobilized on ice and dissected in cold NaCl 0.9% (w/v) for removal of the anterior midgut (AM) and posterior midgut (PM). Preparation of AM and PM fractions was performed according to a modification of the protocols of (Houseman and Downe, 1983a; Houseman and Downe, 1983b) to minimize cell disruption. Due to the fragility of the PM, different protocols were used for each midgut section. To separate the tissues from soluble fractions, each PM was gently homogenized in 100 µL of PBS, using a manual pestle for microtubes (SIGMA, Product N° Z359971) and centrifuged at 4°C for 5 min at 5,000 x g. The supernatants were collected (Posterior Midgut Contents, PMC), and the tissue precipitates were resuspended in 100 µL of PBS (Posterior Midgut Tissues, PMT). Each AM was disrupted and vortexed in 100 µL saline for 5 s, 3 times. The tissues were removed from the tubes with tweezers, and the washings were pooled (final volume 300 μL, Anterior Midgut Contents, AMC). The tissues were immediately homogenized in 100 µL of PBS (Anterior Midgut Tissues, AMT), using a mechanical homogenizer for microtubes (SIGMA, Product N° Z359971).

### 2.6 Enzymatic assays

For enzymatic assays, 25 µL of the sample at appropriate dilution was mixed with 74 µL of 0.2 M sodium acetate buffer pH 4.5 and 1 µL of 4-methylumbelliferyl α-L-fucopyranoside stock solution. Stock solutions of the substrate were prepared in dimethyl sulfoxide (DMSO) at 10 mM. The hydrolysis product was determined by measuring fluorescence at λEx = 355 nm (excitation) and λEm = 460 nm (emission) wavelengths, at 30°C in a spectrofluorometer Spectramax Gemini XPS (Molecular Devices). We measured fluorescence intensity for 60 min with readings at every minute (Profeta et al., 2017).

A standard curve of methylumbelliferone for converting fluorescence data into units of enzymatic activity was made. Activities were expressed in micro units (µU) per insect. One unit of activity (U) corresponds to the amount of enzyme capable of hydrolyzing 1 µmol of glycosidic bonds in 1 min (NC-IUB, 1979). The amount of protein in the samples was determined with the Coomassie Blue G reagent (Bradford, 1976; Felsenstein, 2008), using egg albumin as a protein standard. Enzyme assays were performed with samples submitted to only one cycle of freezing and thawing after their preparation, and protein measurements were performed with samples submitted to a maximun of 3 freezing/thawing cycles.

### 2.7 Effect of freezing on enzyme activity

Soluble fractions were prepared as mentioned above for AM and PM, salivary glands (GS) and hindgut (HG). All samples were homogenized in the same way as PMT. The homogenate was collected and divided into 50 µL aliquots, totaling four aliquots, which were subjected to a different number of freezing/thawing cycles. After the freezing/thawing cycles (0–3 cycles), enzyme activity was measured in all aliquots.

### 2.8 Statistical analysis

Graphs and data analysis were performed with the software GraphPad Prism version 6.04, using one-way Anova with Tukey’s multiple comparisons test, sign test, Spearman correlation test, unpaired *t*-test, Kruskal Wallis test, Mann-Whitney U test, or Fisher’s exact test when appropriate. All results with *p*-value < 0.05 were classified as statistically significant and specified in the graphs.

## 3 Results

### 3.1 Enzyme stability

Before proceeding to the enzymatic assays of samples from insects fed with artificial diets or blood fractions, we tested if the α-L-fucosidase activity was stable after several freezing and thawing cycles. No significant loss of activity was observed between fresh and frozen samples, which were assayed after freezing and thawing cycles (Supplementary Figure S1). Despite the observation of activity changes in most samples after freezing and thawing, it was not possible to confirm a clear pattern of change due to these treatments (Supplementary Table S1). Due to this, we decided to assay the activity of all samples after only one cycle of freezing and thawing. This allowed us to assay samples at different days after feeding or dissection of the insects.

### 3.2 Production of α-L-fucosidase after blood feeding

#### 3.2.1 Defibrinated blood

We performed an initial set of experiments to verify if α-L-fucosidase was increased in the different fractions of the midgut of *Rhodnius prolixus* after blood feeding. Concomitantly, we intended to test if different blood fractions were responsible for this physiological response. For that, *R. prolixus* male adults were fed with defibrinated rabbit blood, which is the regular source of blood for the maintenance of our insect colony, and plasma and cell fractions of the same blood source in an artificial apparatus. To monitor for artifacts due to the possibility of different rates of ingestion or even rejection of these food sources, the insects were weighed individually before and after feeding. As expected, insects fed with control blood showed a significant increase in weight after feeding when compared to insects before feeding. The same phenomenon was observed after feeding the insects with plasma and cell fractions. No significant differences between the weights of the unfed insects of the three groups considered were observed. However, analysis of insect weights after feeding showed a significant difference between at least two of these groups. Interestingly, no significant differences were observed when comparing control insects to the experimental ones fed with plasma or cell fractions, but a significant difference was observed between plasma and cell fractions, indicating that the mean volume of plasma ingested was 15% lower than the volume of ingested cell fraction (Supplementary Figure S2A; Supplementary Table S2A).

In the course of the series of experiments with defibrinated blood, we realized that some degree of hemolysis happened during the preparations of blood fractions (data not shown). It was impossible to get samples with no hemolysis, probably because it happened throughout the defibrination treatment. Due to this, we restricted this series of experiments to only two biological replicates, which severely limited the kind of statistical analysis that could be performed with this data (see below). However, the data obtained with defibrinated blood indicated for us the general trends of our system, as can be seen below.

Having that ingestion was not different among the different treatments and the control group, we measured the α-L-fucosidase activity in these insect groups in four different midgut fractions: anterior midgut contents (AMC), anterior midgut tissues (AMT), posterior midgut contents (PMC), and posterior midgut tissues (PMT), 5 days after the ingestion of food.

Considering the insects fed with whole defibrinated blood, data suggest that AMC was the only fraction with activity after feeding (AF) different from the activity observed in the same midgut fraction taken from insects before feeding (BF). In the other fractions (AMT, PMC, and PMT) data indicate no differences between the activities after feeding and before feeding (control group in Supplementary Figure S2B; Supplementary Table S2B). These data suggest that AMC was the only fraction where α-L-fucosidase was increased in the midgut of *R. prolixus* after ingestion of whole blood. Considering the feeding with blood fractions, trend suggests that insects fed with plasma showed α-L-fucosidase activities in the AMC higher than BF activities. However, no indication of differences were observed between the α-L-fucosidase activities in the other gut fractions of insects fed with plasma and insects before feeding. We also observed a suggestion of difference in the AMT when comparing insects fed with plasma to insects fed with whole blood (Supplementary Figure S2B; Supplementary Table S2B).

Feeding with cell fraction resulted in no significant increase in α-L-fucosidase activities in any gut fraction when compared to insects before feeding. Interestingly, in the AMT the activity of insects fed with cell fraction was significantly lower than before feeding. When comparing to control insects feed with whole blood, insects fed with the cell fraction showed no significant differences in activity in any gut fraction (Supplementary Figure S2B; Supplementary Table S2B). These preliminary results suggest that α-L-fucosidase is primarily increased in the AMC after blood feeding, and that the plasma was the fraction responsible for this gut response. However, the limitation of the data (especially the low number of replicates) and problems with hemolysis in the defibrinated blood led us to explore better experimental setups (see below).

In these experiments, we decided to describe α-L-fucosidase as units of enzyme activity per insect, because specific activity measurements (as mU/mg protein) were misleading due to the high protein content of the diet. We observed a significant increase in the AMC protein concentrations after feeding with defibrinated blood (control), plasma and cell fractions, and in the AMT after feeding with plasma or cell fraction. No significant changes were observed in the PMC, and PMT (Supplementary Figure S3; Supplementary Table S3).

#### 3.2.2 Citrated blood

**FIGURE 1:**
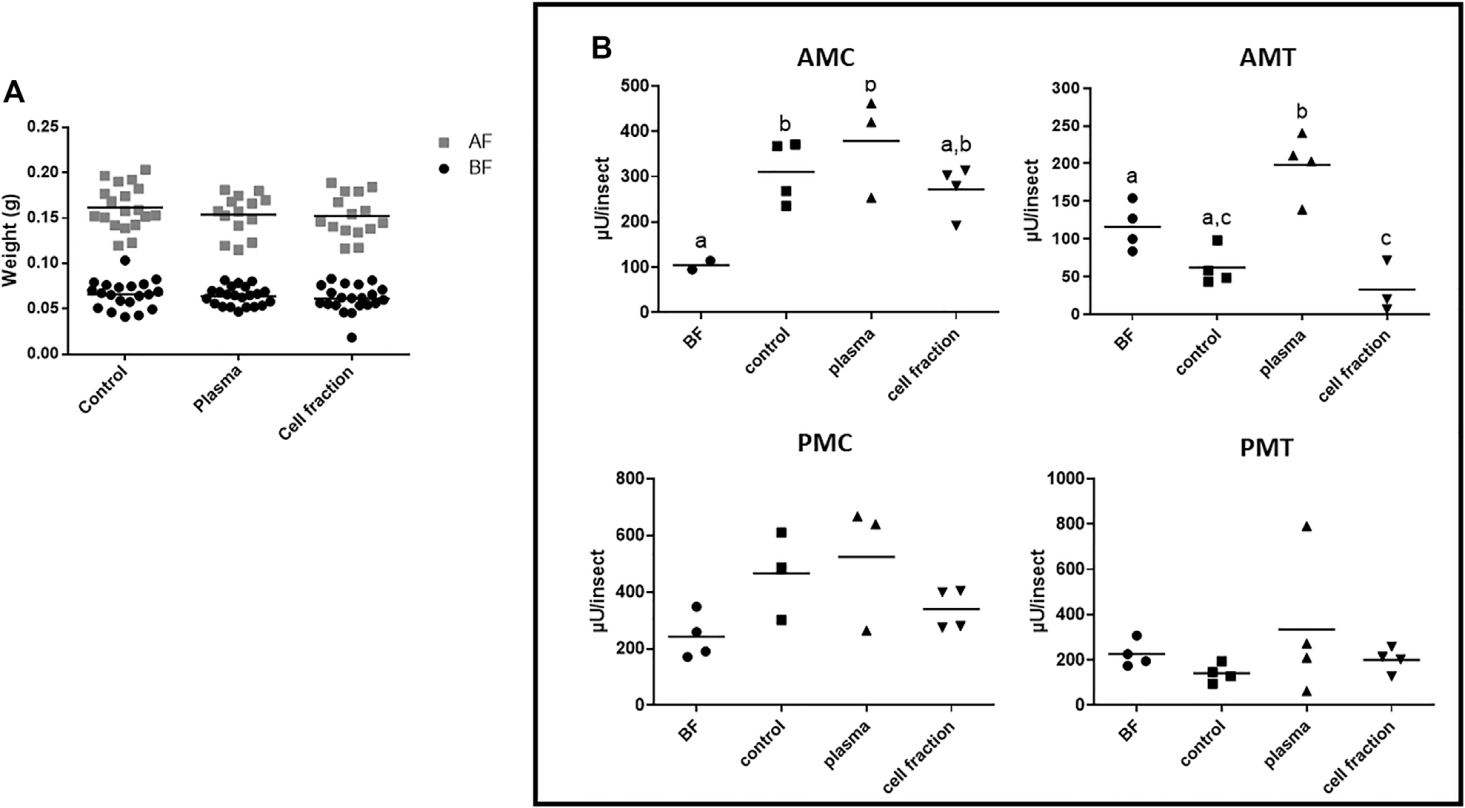
*Rhodnius prolixus* weight and midgut α-L-fucosidase activities before and after feeding with citrated blood or different blood fractions. **(A)** Weights of individual insects before and after feeding. BF: Before feeding (black circles), AF: After feeding (gray squares). N = 20 insects in each group/condition. **(B)** α-L-fucosidase activity before feeding (BF) or 5 days after feeding, in different midgut fractions. AMC: anterior midgut contents; AMT: anterior midgut tissues; PMC: posterior midgut contents; PMT: posterior midgut tissues. BF: before feeding corresponds to insects that were starved for 40 days. The groups marked with different letters (a,b,c) correspond to sets of values that are statistically different from each other (see Supplementary Table S4). n = 4 pools of 5 insects each. Longitudinal bars show the arithmetic mean of each data group.

Due to the problems observed with defibrinated blood (see above), we ran a second series of experiments, using citrated rabbit blood as the feeding source, to discard interferences in the results above obtained with plasma from contamination with cell contents, and *vice versa*. Insects fed with citrated blood or its fractions showed a significant increase in weight, comparing the weights of insects before and after blood feeding (Figure 1A; Supplementary Table S4A), and the weight of insects fed with both plasma or cell fraction showed no significant difference when compared to controls fed with the whole citrated blood (Figure 1A, Supplementary Table S4A). In this respect, we observed that the artificial system with citrated blood was very well accepted by the insects, allowing the direct analysis of α-L-fucosidase gut activities.

The assay of different midgut fractions after feeding with citrated whole blood showed that the only fraction where the activity of α-L-fucosidase was significantly higher than the observed in insects before feeding was the AMC (Figure 1B, Supplementary Table S4B). No significant differences between the activity after and before feeding were observed in the AMT, PMC, and PMT (Figure 1B, Supplementary Table S4B). This reinforces the observation that the production of α-L-fucosidase after blood feeding results in activity increase primarily in the AMC. Feeding with plasma generated similar results, with activities significantly higher than the observed in insects before feeding in AMC (Figure 1B, Supplementary Table S4BB), but no significant difference in the AMT, PMC and PMT (Figure 1B, Supplementary Table S4B). Besides that, no significant changes were observed after feeding with the cell fraction in the AMT, PMC, and PMT, when we compared these activities with those from non-fed insects (Figure 1B, Supplementary Table S4B), and in the AMC, the data suggested an increase in activity, but the difference was not statistically significant (Figure 1B, Supplementary Table S4B).

**FIGURE 2:**
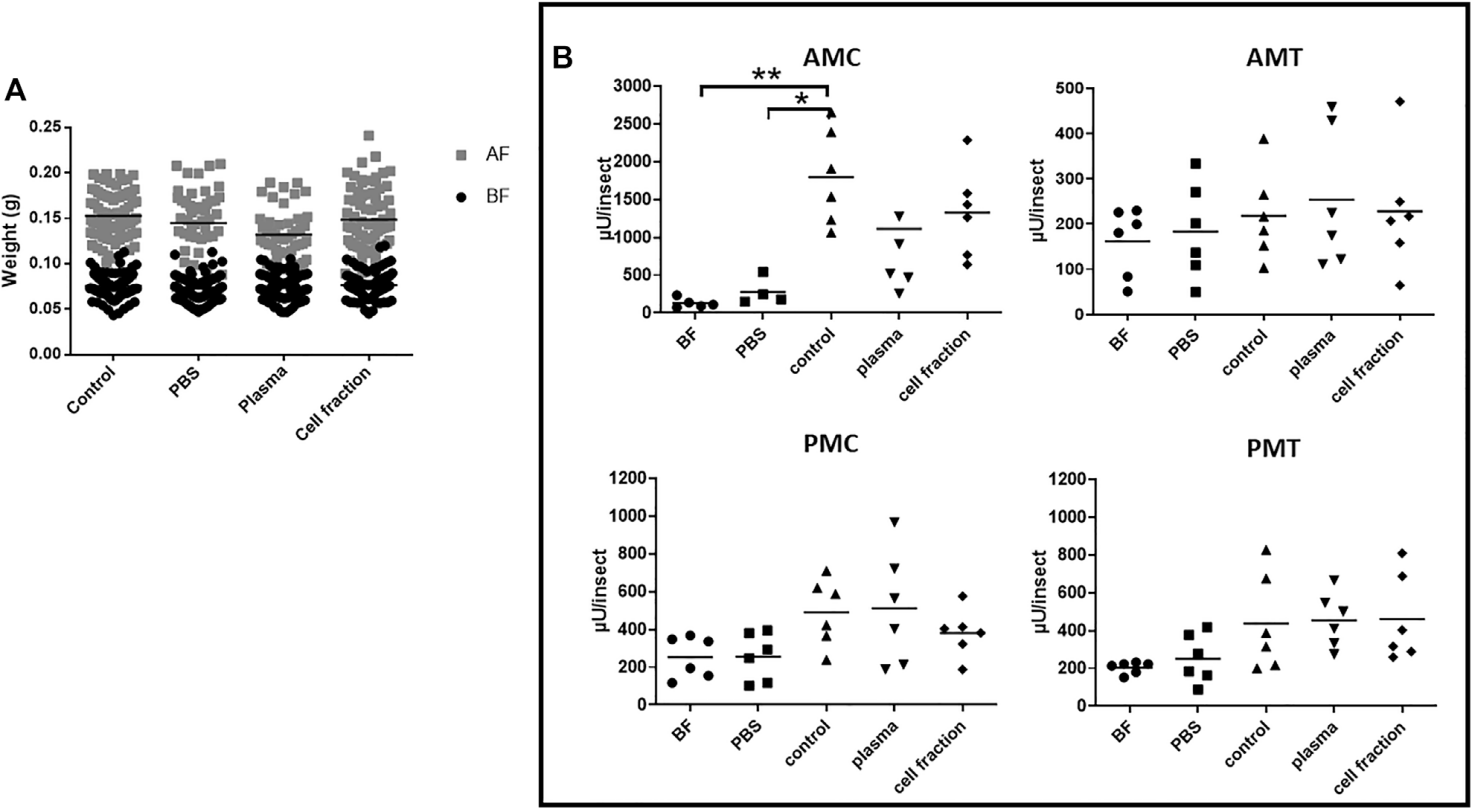
*Rhodnius prolixus* weight and midgut α-L-fucosidase activities before and after feeding with heparinized blood or different blood fractions. **(A)** Weights of individual insects before and after feeding. BF: Before feeding (black circles), AF: After feeding (gray squares). PBS: Phosphate buffered saline (pH 7.2). N = 100 insects in each group/condition. **(B)** α-L-fucosidase activity before feeding or 5 days after feeding, in different midgut fractions. AMC: anterior midgut contents; AMT: anterior midgut tissues; PMC: posterior midgut contents; PMT: posterior midgut tissues. BF: before feeding corresponds to insects that were starved for 40 days. The groups marked with asterisk correspond to sets of values that are statistically different from each other (see Supplementary Table S6). n = 6 pools of 5 insects each. Longitudinal bars show the arithmetic mean of each data group.

However, when we compare the results obtained with insects fed with defibrinated blood to those obtained with citrated blood, we observed that the AMC of insects fed with citrated blood showed activities in a range significantly lower (around 300 µU/insect) than the observed in insects fed with defibrinated blood (around 900 µU/insect Figure 1B; Figure 2B). This result suggested that citrate may inhibit the α-L-fucosidase production, or that the general composition of blood may affect significantly the general digestive physiology, resulting in different patterns of production of enzymes in the midgut. Importantly, there were a significant increases in the AMC and AMT protein values after feeding with citrated blood, but the same changes were not observed in insect fed with plasma, and the increases observed in insects fed with the cell fraction were not statistically significant (Supplementary Figure S4, Supplementary Table S5). This last issue may be due to the low number of replicates and high errors in the cell fraction group. No significant changes in the protein amounts were observed in the PMC and PMT after any treatment (Supplementary Figure S4, Supplementary Table S5).

#### 3.2.3 Heparinated blood

To test the hypothesis of citrate as an inhibitor of α-L-fucosidase production, we decided to run the third series of experiments, this time offering heparinated blood to the insects. As observed before, there was a significative intake of heparinated blood and their plasma and cell fractions, measured as individual weights significantly higher than those observed before feeding (Figure 2A, Supplementary Table S6A). Besides that, there were no significant differences between the intake of whole blood and the cell fraction (Figure 2A, Supplementary Table S6A). However, insects fed with plasma showed weights after feeding that were significantly lower than the controls (Figure 2A, Supplementary Table S6A), with a mean weight after feeding 14% lower. In this series of experiments, we included a group of insects fed with PBS, to evaluate if the distension alone of the body had any effect on α-L-fucosidase activity. The insects fed with PBS successfully, with no significant difference in weight increase when compared to the other groups (Figure 2A, Supplementary Table S6A).

Feeding with PBS did not result in α-L-fucosidase significantly different from the observed in insects before feeding in any of the considered fractions of the midgut, AMC, AMT, PMC, and PMT (Figure 2B; Supplementary Table S6B). Feeding with heparinated blood resulted in a significative increase in α-L-fucosidase activity in the AMC and PMC when compared to non-fed insects (Figure 2B, Supplementary Table S6B), but no increase was observed in the AMT, and PMT (Figure 2B, Supplementary Table S6B). Significant increases of α-L-fucosidase activity in the AMC and PMT were observed after feeding the insects with plasma from heparinated blood when compared to non-fed insects, but no significant changes were observed in the AMT and PMC (Figure 2B and Supplementary Table S6B). Feeding with the cell fraction of heparinated blood resulted in outcomes similar to the observed with plasma, namely, significant increases in AMC and PMT, and no significant changes in the AMT and PMC (Figure 2 and Supplementary Table S6B). Interestingly, the activity produced after feeding with heparinated blood in the AMC (range around 2000 µU/insect) was much higher than the observed with defibrinated (around 900 µU/insect) or citrated blood (around 300 µU/insect) (Supplementary Figure S2, and Figure 1; Figure 2). Importantly, this experiment confirmed that the production of α-L-fucosidase after blood feeding occurred mostly in the AMC. Regarding protein amounts, feeding with heparinated blood resulted in significant increases in the AMC and PMC, with no significant changes in the AMT and PMT when compared to unfed controls or controls fed with PBS (Supplementary Figure S5 and Supplementary Table S7). Feeding with plasma resulted in a significant increase only in the AMC, with no significant changes in the other gut fractions when compared to unfed controls or controls fed with PBS (Supplementary Figure S5 and Supplementary Table S7). However, the AMC protein amounts in insects fed with plasma were different from those observed in insects fed with whole heparinated blood or the cell fraction (Supplementary Figure S5 and Supplementary Table S7). This suggested a faster protein passage through the gut after feeding with plasma only. Feeding with the cell fraction resulted in effects that were similar to the whole blood, except for a significant higher protein content in the AMT when compared to non-fed or PBS controls (Supplementary Figure S5 and Supplementary Table S7).

**FIGURE 3:**
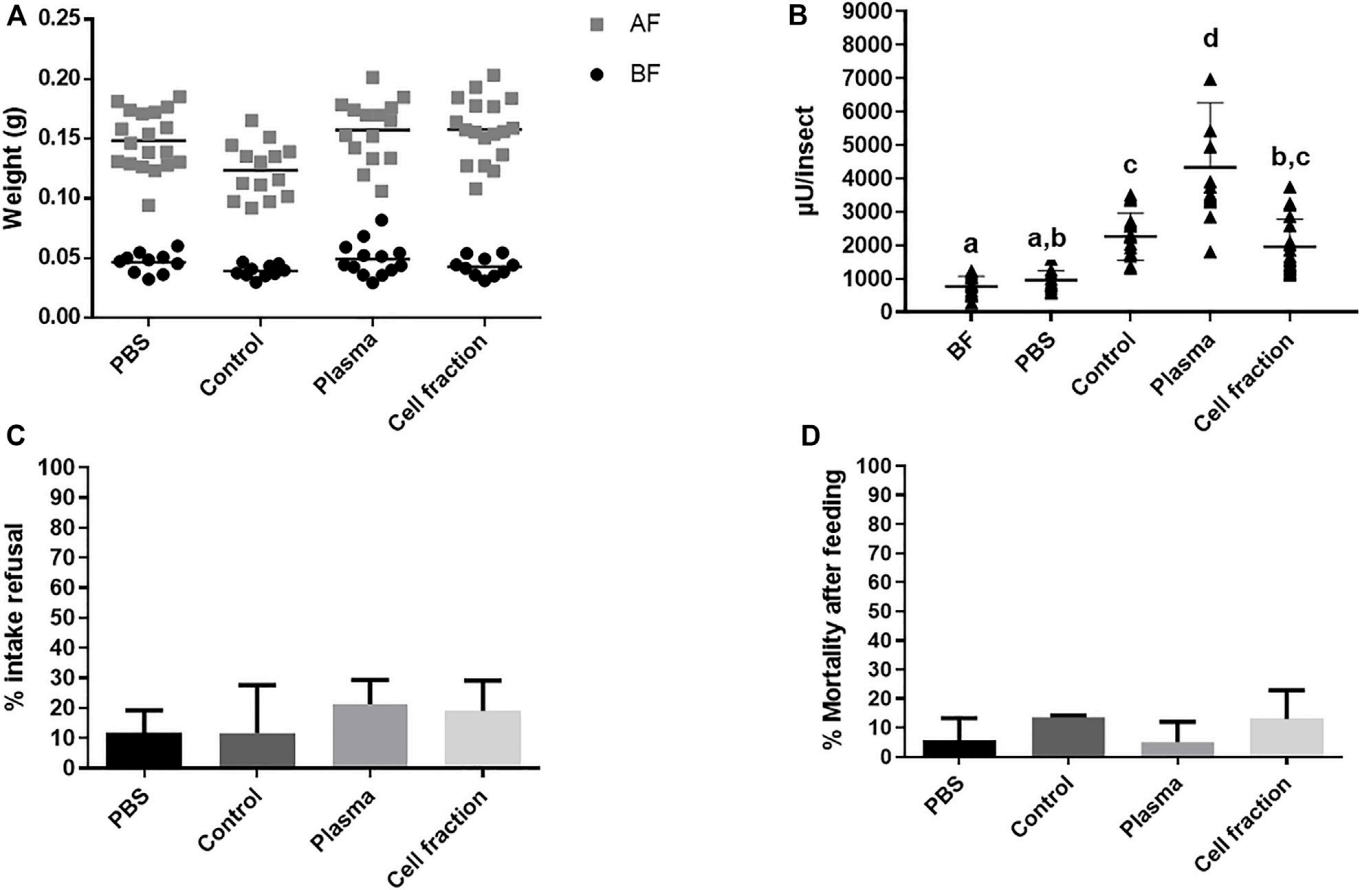
*Rhodnius prolixus* weight and α-L-fucosidase activities before and after feeding with heparinized blood (control) or different blood fractions (Plasma or Cell fraction; Cell fraction was washed 3 times). **(A)** Weights of individual insects before and after feeding. BF: Before feeding (black circles), AF: After feeding (gray squares). PBS: Phosphate buffered saline (pH 7.2). N = 20 insects in each group/condition. **(B)** α-L-fucosidase activity before feeding or 5 days after feeding, in the anterior midgut contents (AMC). BF: before feeding corresponds to insects that were starved for 40 days. The groups marked with different letters (a,b,c) corresponded to sets of values that are statistically different from each other (see Supplementary Table S8). n = 15 insects individually measured. **(C)** *R. prolixus* refusal rate to different meals and blood fractions. Percentage of insects that did not engorge after being exposed to different feeds. **(D)** *R. prolixus* mortality rate. Percentage of engorged insects that died until 5 days after feeding. For (C) and (D), N = 3 replicates with 20 insects each. Longitudinal bars in (A) and (B) show the arithmetic mean of each data group. Error bars in (B-D) correspond to the Standard Deviation (SD) in each group.

The observation of similars increase of activity after feeding with plasma or cell fractions from heparinated blood, contrasting with the results obtained with defibrinated or citrated blood, let us to think that in this case, more washings of the cells might be necessary to avoid cross contamination or interference between fractions and that more experimental replicas would be necessary to describe the effects of this blood source due to higher dispersion of data. A new series of experiments was performed, adding additional washes of cells before reconstitution of the cell fraction, and including more experimental replicas. This time, the analysis focused on the AMC activities only. As observed before, significant increases in weight were observed in insects fed with heparinated whole blood (control), PBS, plasma, and cell fractions, when compared to insects before feeding in their respective groups (Figure 3A and Supplementary Table S8A). No significant differences between groups were observed, with the exception of an approximately 20% lower weight in the control fed group, when compared to insects fed with PBS, Plasma or Cell Fraction (Figure 3A, Supplementary Table S8A). Looking at α-L-fucosidase activities in the AMC, a significant increase in activity was observed in the groups fed with whole heparinated blood and plasma, when compared to the groups before feeding and fed with PBS (Figure 3B, Supplementary Table S8B). The insects fed with cell fraction showed a high dispersion of data, and despite showing a higher activity than the non fed controls, their activities did not significantly differ from PBS controls, as well as to insects fed with whole blood (Figure 3B, Supplementary Table S8B). Noteworthy, the activity observed after feeding with plasma was higher than that observed after feeding with whole blood or the cell fraction (Figure 3B, Supplementary Table S8B). Feeding with whole blood resulted in a significant increase in the AMC protein contents, when compared to non-fed and PBS controls, but a similar significant increase after feeding with plasma or the cell fraction was not observed (Supplementary Figure S6A, Supplementary Table S9A).

From this point we started to monitor the refusal rate and the mortality of insects after feeding, which would be indicators of aggressive or stressful conditions. We decided to do that due to the inclusion of the PBS group, and because these parameters were important for the testing of the inclusion of individual components or molecules in the food (see below). As expected, in all groups the refusal rate was very low (10%–20%) with no differences between groups (Figure 3C, Supplementary Table S8C). All groups showed very low mortality levels (below 20%) with no significant differences observed between the treatments (Figure 3D and Supplementary Table S8D).

#### 3.2.4 Feeding with isolated proteins and carbohydrates

**FIGURE 4:**
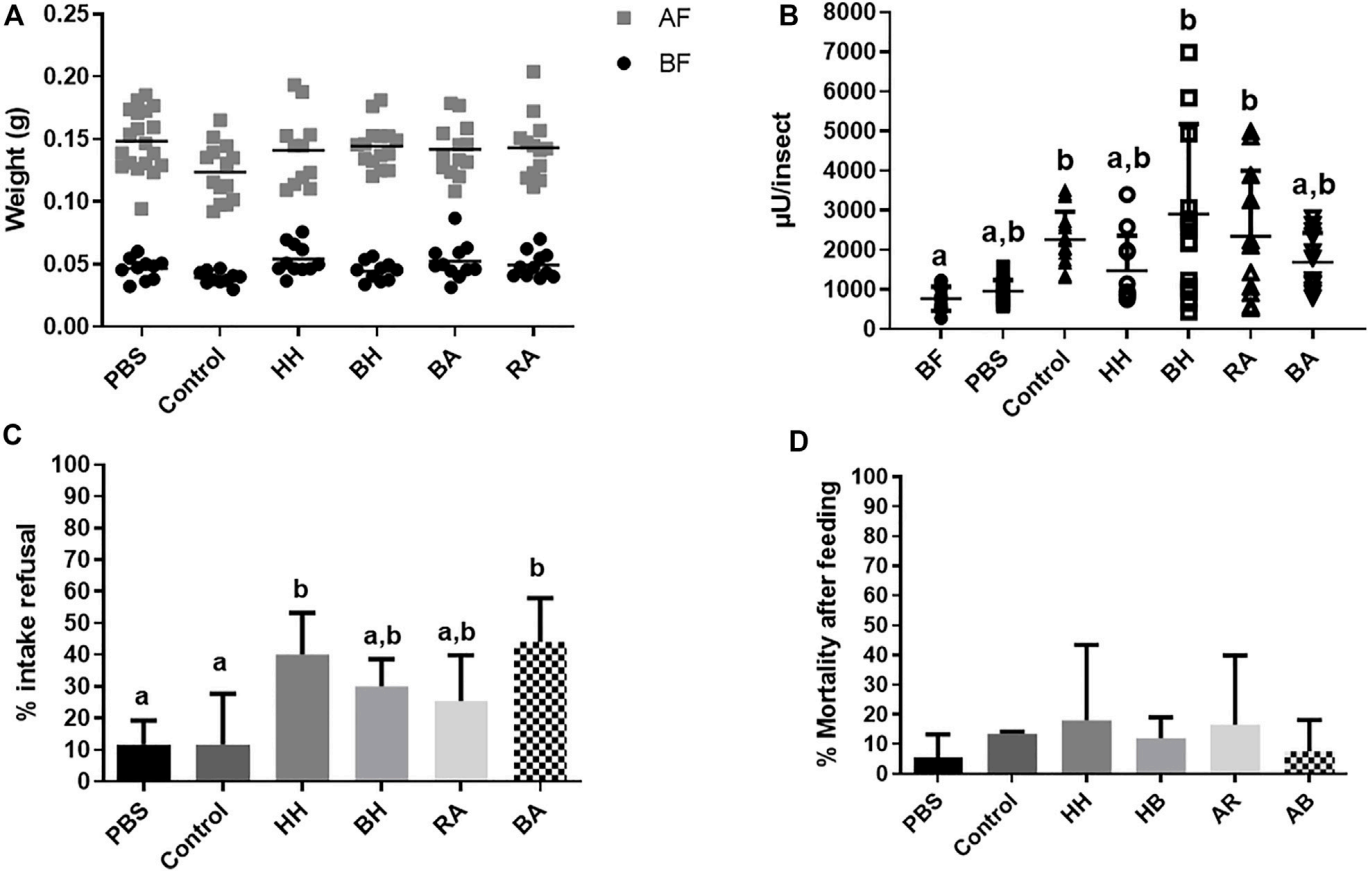
*Rhodnius prolixus* weight and α-L-fucosidase activities before and after feeding with heparinized blood (control) or different proteins. **(A)** Weights of individual insects before and after feeding. BF: Before feeding (black circles), AF: After feeding (gray squares). N = 20 insects in each group/condition. **(B)** α-L-fucosidase activity in the anterior midgut contents (AMC) before feeding (BF) or 5 days after feeding. BF: before feeding corresponds to insects that were starved for 40 days. PBS: Phosphate buffered saline (pH 7.2). HH: Human Hemoglobin; BH: Bovine Hemoglobin; RA: Rabbit Albumin, BA: Bovine Albumin. The groups marked with different letters (a,b,c) correspond to sets of values that are statistically different from each other (see Supplementary Table S10). n = 12 individual insects. **(C)** *R. prolixus* refusal rate to feeding on different control or protein solutions. Percentage of insects that did not engorge after being exposed to different meals. N = 3 group replicates with 20 insects each. **(D)**: *R. prolixus* mortality rate 5 days after feeding on the different meals tested. N = 3 group replicates with 20 insects each. Longitudinal bars in (A) and (B) show the arithmetic mean of each data group. Error bars in (B-D) correspond to the Standard Deviation (SD) in each group.

Next, we performed a series of experiments to verify if the production of α-L-fucosidase in the AMC, observed after feeding with defibrinated, citrated, and heparinated blood, and with the plasma fraction of all these feeds, was related to the intake of particular blood components. The first component we tested were two different blood proteins, hemoglobin and albumin. We chose these two proteins because they are representative components of the two blood fractions studied here, respectively the cell and plasma fractions. Besides that, we decided to use blood proteins from different animal origins (human, bovine, and rabbit), to test if some specific structure from the food proteins may result in different responses. Offering a solution of these proteins (human hemoglobin, bovine hemoglobin, bovine albumin, and rabbit albumin) resulted in individual weights after feeding that do not significantly differ from those observed with PBS or whole heparinized blood (Figure 4A and Supplementary Table S10A), showing that the feeds were successful. In terms of α-L-fucosidase, only the AMC of insects fed with bovine hemoglobin and rabbit albumin showed significantly higher activities than non-fed insects (Figure 4B, Supplementary Table S10B), but with values more dispersed than the observed in the controls fed with heparinated blood. However, insects fed by human hemoglobin and bovine albumin, despite having mean activity values that were higher than non-fed or PBS fed controls, showed no significant differences from the controls above (Figure 4B, Supplementary Table S10B). It is important to notice the high dispersion of values in these groups, which accounted for the superposition of results. To rule out the toxic effects of these protein feeds, we evaluated the intake refusal and mortality after feeding in all groups. The insects that were offered protein diets showed higher refusal rates (25%–40%) than the controls (10%). Insects offered human hemoglobin and bovine albumin showed significant higher refusal rates than controls, but no significant difference was observed between controls and insects offered bovine hemoglobin and rabbit albumin (Figure 4C, Supplementary Table S10C). We also observed a mortality rate around 20% in insects fed with human hemoglobin or rabbit albumin, but no significantly different to the other groups, which showed mortalities around 5%–10% (Figure 4D, Supplementary Table S10D). Surprisingly, in terms of AMC protein amounts, there were no significant differences between insects fed with Human or Bovine Hemoglobin and Albumin and the non-fed or PBS controls (Supplementary Figure S6B, Supplementary Table S9B). In this set of experiments, only the blood fed control showed a protein amount significantly higher than non-fed or PBS controls (Supplementary Figure S6B, Supplementary Table S9B).

**FIGURE 5:**
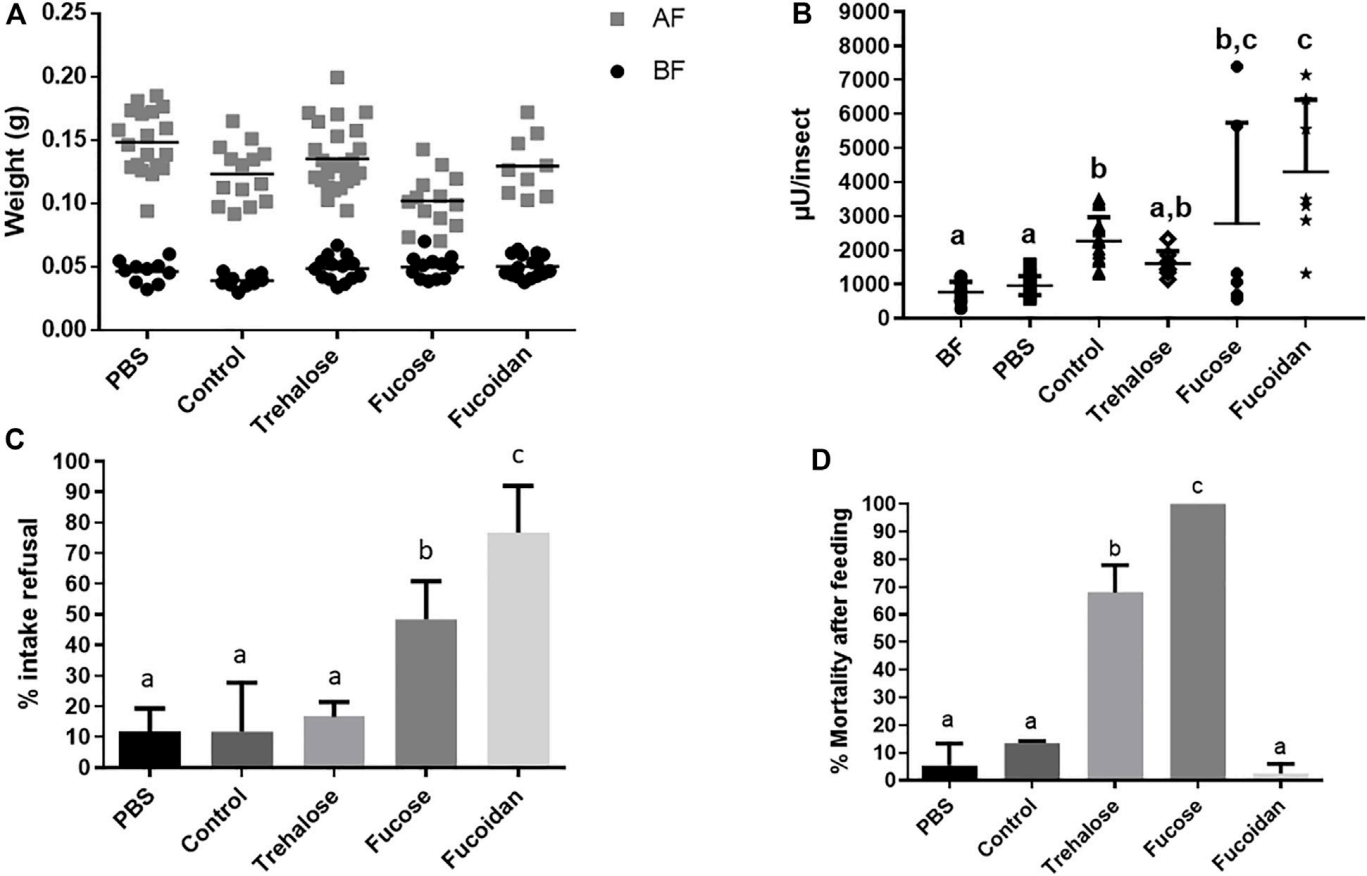
*Rhodnius prolixus* weight and α-L-fucosidase activities before and after feeding with PBS, heparinized blood (Control) or different sugar solutions. **(A)** Weights of individual insects before and after feeding. BF: Before feeding (black circles), AF: After feeding (gray squares). N = 20 insects in each group/condition. **(B)** α-L-fucosidase activity in the anterior midgut contents (AMC) before feeding (BF) or 5 days after feeding. BF: before feeding corresponds to insects that were starved for 40 days. PBS: Phosphate buffered saline (pH 7.2). The columns marked with different letters (a,b,c) correspond to sets of values that are statistically different from each other (see Supplementary Table S11). N = 8 individual insects. **(C)** *R. prolixus* refusal rate to feeding on different control or sugar solutions. Percentage of insects that did not engorge after being exposed to different meals. N = 3 replicates with 20 insects each. **(D)** *R. prolixus* mortality rate 5 days after feeding on the different meals tested. N = 3 replicates with 20 insects each. Longitudinal bars in (A) and (B) show the arithmetic mean of each data group. Error bars in (B-D) correspond to the Standard Deviation (SD) in each group.

The second type of molecules we added to the diet were different carbohydrates, to test if the α-L-fucosidase production in the AMC could be stimulated by some blood carbohydrate. We initially chose L-fucose, representing the fucose moiety of blood glycoproteins. Due to toxicity issues (see below), we added a group fed with fucoidan and, also, to rule out a generic effect due to ingestion of sugars, we included treatment with trehalose. Initially, we were able to observe significant weight increases in all tested groups, showing that all groups were able to feed (Figure 5A, Supplementary Table S11A). In this set of experiments, before feeding, the control group had a mean weight lower (by 16%–20%) than the other groups, and, after feeding, the groups fed with PBS and Fucose showed mean weights that were bigger (by 10%–45%) and smaller (by 15%–25%) than the other groups, respectively (Figure 5A, Supplementary Table S11A). In respect to AMC α-L-fucosidase levels, insects fed with L-fucose and fucoidan showed activities significantly higher than the unfed and those fed with PBS, similar to controls fed with heparinated blood (Figure 5B, Supplementary Table S11B). The activity of insects fed with trehalose was not significantly different from the activity of PBS fed and unfed controls but, due to a higher dispersal of values, was also not different to the controls fed with blood (Figure 5B, Supplementary Table S11B). The data suggests that the L-fucose moiety is sufficient to elicit AMC α-L-fucosidase production. However, these data need to be considered with some caution because a significant number of insects refused to take the L-fucose (50%) and fucoidan (75%) meals, in proportions that were significantly higher than those observed in the PBS and heparinated blood fed controls, around 10% (Figure 5C, Supplementary Table S11C). A slightly higher proportion of insects refused trehalose (15%), but the values were not significantly different from the observed in the controls above (Figure 5C, Supplementary Table S11C). Besides that, ingestion of trehalose and L-fucose were highly deleterious—among the insects that fed, respectively 65% and 100% died after feeding, both proportions significantly higher than the observed in PBS and heparinated blood fed controls, around or below 10% (Figure 5D, Supplementary Table S11D). Mortality after feeding with fucoidan was very low, but was not significantly different than the observed in the controls above (Figure 5D, Supplementary Table S11D). In terms of AMC protein amounts, ingestion of trehalose or fucoidan did not result in a significant increase when compared to non-fed or PBS fed controls (Supplementary Figure S6C, Supplementary Table S9C). In this set of experiments, only the blood feed resulted in a significant increase in AMC protein amounts when compared to non-fed or PBS controls (Supplementary Figure S6C, Supplementary Table S9C), and no protein quantitation results were obtained from insects fed with L-fucose, due to the death of all insects (see above).

## 4 Discussion

Blood feeding in artificial systems is a widely used tool for the study of several aspects of physiology in hematophagous insects. In particular, several works were published about *R. prolixus* digestion and its interaction with *T. cruzi* using this tool. For example, several works on the characterization of midgut enzymes, including proteases (Ferreira et al., 1988a; Terra et al., 1988), carbohydrases (Ribeiro and Pereira, 1984), lipases (Grillo et al., 2007), phosphatases (Terra et al., 1988), salivary proteins (Corte-Real et al., 2011), and other intestinal proteins as lectins (Araujo et al., 2021). Immunological studies and studies on the interaction of *R. prolixus* with gut bacteria or parasites recurrently used artificial apparatus for blood feeding, to standardize the initial inoculum and status of microorganisms at the time of infection (Batista et al., 2020; 2021).

When recurring to an artificial system for blood feeding, blood clotting must be inhibited, and the most common strategies for that are defibrination or the addition of citrate or heparin. Blood defibrination can result in hemolysis, as observed in this work. However, it is cheap and reliable strategy for feeding animals that require high amounts of blood, such as kissing bugs, and it was the strategy of choice for large scale rearing of *R. prolixus* in our group for several years (Azambuja and Garcia, 1997). The use of this strategy in works characterizing the production of *R. prolixus* midgut enzymes after blood feeding is of special interest. This system was used successfully to describe the production pattern of glycosidases (Ribeiro and Pereira, 1984; Gama et al., 2022) and proteases (Henriques et al., 2021), but because these works fed the insect only with whole blood, no reference to hemolysis was made. In a particular work, *R. prolixus* was fed with whole defibrinated blood, its plasma and cell fractions were diluted with PBS, and a secretatogue pattern of stimulation was demonstrated by their dilutions, with no reference to hemolysis (Garcia and Garcia, 1977).

Citrated blood was used by different groups in several contexts, especially for studies of *T. cruzi* infection (Silva-Cardoso et al., 2018). However, citrate can alter the course of digestion or development, due to the presence of digestive enzymes or other proteins that depend on calcium ions for their activity. A recent report showed an important negative impact of citrate on *R. prolixus* physiology, with impairment of oviposition and egg hatching (Silva-Cardoso et al., 2018). Our results showed a strong reduction of midgut α-L-fucosidase after feeding with citrated blood, but it is not clear if this is caused by direct inhibition of the enzyme or by indirect physiological effects resulting in lower levels of α-L-fucosidase. Considering that we did not find any record of inhibition of α-L-fucosidases by citrate, the second hypothesis seems more likely, but this certainly deserves more investigation.

In our experiments, heparinated blood resulted in the highest α-L-fucosidase activities. Furthermore, heparin has been recommended as the anticoagulant of choice for *R. prolixus* feeding experiments, due to minimal physiological impacts and no interference in biochemical and molecular biology assays (Silva-Cardoso et al., 2018). However, despite differences between the general digestive patterns of *R. prolixus* fed in these different blood treatments are not expected, this issue was never investigated in detail. In this respect, we strongly recommend heparinated blood for further characterization of enzyme activities in *R. prolixus* fed by artificial systems.

Another concept we were able to explore about blood feeding was the possible impact of the animal source in the production of α-L-fucosidase. This was tested using feeding with specific proteins (hemoglobin or albumin) from different animals, namely, bovine, rabbit, or human. The source of the protein had no impact on the effect observed. Interestingly, it was already demonstrated that blood source has a very strong effect on *R. prolixus* development and reproduction (Gomes et al., 1990). Considering that we saw no changes in the activity of α-L-fucosidase, it is likely that the effect of blood source is not related to differential patterns of enzyme production. However, this hypothesis needs more confirmation with the study of different enzymes.

According to our results, the main site of production of α-L-fucosidase in *R. prolixus* midgut after blood feeding was the AMC. In this respect, the enzyme must be somehow secreted to the midgut lumen. Preliminary analysis showed that *R. prolixus* genome contains only one canonical α-L-fucosidase coding gene belonging to Glycoside Hydrolase Family 29 (RPRC007504; Souza, 2016), and that the coding region of this gene has a putative signal peptide (data not shown). This suggests that *R. prolixus* midgut α-L-fucosidase follows the exocytic secretory pathway, as observed for other digestive enzymes of this insect (Ferreira et al., 1988b). However, it is important to mention that for Hemiptera, secretion of midgut enzymes to the lumen involves the production of double membrane vesicles, to secrete the components of the perimicrovillar membrane together with molecules targeted to the luminal contents (Ferreira et al., 1988b). Secreted digestive α-L-fucosidases were already described in spiders (Perrela et al., 2018a), ticks (Moreti et al., 2013), and molluscs (Perrela et al., 2015), but a putative signal peptide was described only for the spider activity. In contrast, there are also reports of α-L-fucosidase adhered to tissue in mollusks (Escobar-Correas et al., 2019).

Our data suggest that the α-L-fucosidase may be considered a secreted digestive enzyme, being produced after a blood meal and majorly secreted to the lumen of the anterior midgut. In a broad sense, insect digestive enzymes can be classified based on their expression pattern, as constitutive or induced proteins (Terra and Ferreira, 1994; Silva et al., 2012). The first pattern is common amongst holometabolous larvae, and the second is common in hematophagous adult dipterans and hemipterans (Terra and Ferreira, 1994). The information available about the pattern of production of insect α-L-fucosidases is very scarce and, for our records, this is the first description of an non-constitutive insect α-L-fucosidase, but this certainly needs more investigation looking to other models. Besides that, it is still not clear what was the mechanism of production but based on the 5-day time frame analyzed in our experiments, it is likely that the activity increase is related to the activation of gene expression. This is certainly a point to be investigated in the future.

The series of experiments performed to determine if there is any ingestion or dietary signal responsible for the production of the AMC α-L-fucosidase suggested an indirect mechanism, with no clear connection to the composition of the food. Initially, we observed that ingestion of PBS alone was not enough to trigger the response, similar to what was observed previously for the production of proteases in the posterior midgut (Garcia and Garcia, 1977). This data suggest that the production was connected to a secretagogue mechanism and, for proteases, that was explored by feeding the insect with blood fractions, namely, the plasma or cell fractions (Garcia and Garcia, 1977). For α-L-fucosidase, we observed a significant increase only after ingestion of the plasma fraction, suggesting that some component of plasma was responsible for it. It is noteworthy that, in this case, we were following the production of a digestive enzyme in a different compartment (anterior midgut) than the used for the protease studies (posterior midgut). This might suggest a common mechanism in both compartments, related to the detection of specific blood components.

However, the observation of a significant activity increase after ingestion of several proteins as albumins from different sources, including non-glycosylated proteins such as hemoglobin, strongly suggests that the direct detection of glycosylated products may not be a key factor for this phenomenon. In this respect, this mechanism may be related to the general nutritional status of the insect, and it would be interesting to examine the effects of amino acids, peptides, and inhibitors of the TOR signaling cascade such as Rapamycin. It was demonstrated that the TOR pathway was involved in important physiological responses of the triatomine midgut after blood feeding, as changes in the production of ROS and mitochondrial metabolism (Gandara et al., 2016).

The observation that ingestion of fucoidan, but not trehalose, resulted in significant increase of α-L-fucosidase, strongly suggests the detection of L-fucose as a step for the production of the enzyme. Unfortunately, it was not possible to test fucose as a stimulant due to the high toxicity of this sugar to the insects. Nevertheless, all the results obtained suggest that production of α-L-fucosidase is an outcome of different convergent stimuli, pointing out the possibility that its increase is connected to a more general response of the gut, which may be triggered by different signals and may include other enzymes as well. In this respect, fine tuning of expression by a secretagogue mechanism, as suggested for proteases, may be restricted to the posterior midgut or may be an artifact due to relatively quicker rates of food digestion and passing along the gut after meals with lower protein content.

In this respect, the lack of correlation between specific food molecules and production of digestive enzymes points to a non-specific and indirect mechanism. Among the physiological cascades that may be involved, beyond the TOR pathway, are the production of ecdysone or even the increase of the gut microbiota that was observed after a blood meal. The hormonal regulation of the production of several components of the midgut, such as the perimicrovillar membrane or enzymes such as phenoloxidase, is well documented (Nogueira et al., 1997; Gonzalez et al., 1999; Genta et al., 2010). In this respect, it would be interesting to test if this response is affected by head ablation or azadirachtin treatment.

The increase in the gut microbiota of *R. prolixus* after a blood meal may be as high as 10,000 times and occurs mainly in the anterior midgut (Azambuja et al., 2004), the specific site of increase in α-L-fucosidase activity. Previous reports verified a very low activity of this enzyme in the blood itself, ruling out that this enzyme is acquired from the meal (Souza, 2016). However, the production of α-L-fucosidase by gut microorganisms may be considereda possibility, as this kind of activity (E.C. 3.2.1.51, 3.2.1.63, 3.2.1.111, or 3.2.1.127) was already reported in several bacteria (Chang et al., 2021). Nevertheless, it is interesting to notice that the time course of production of α-L-fucosidase (Souza, 2016) has some superposition with the bacterial growth described for the anterior midgut (Azambuja et al., 2004). In this respect, the effects of feeding with antibiotics will be a straightforward tool for testing this hypothesis. It is important that in other models, cell fucosylation was increased as a response to infection stress in the gut, leading to a fucose content that may be a substrate for microorganisms (Minton, 2014).

Another issue that must be considered is that the lack of specificity observed for the production of α-L-fucosidase makes the definition of the functional role or substrate identity for this enzyme elusive, which might be clarified if we had seen a correspondence for a particular molecule, or a more specific secretagogue mechanism. In this way, it is important to reinforce that our assays used a synthetic substrate, with no clear correspondence to natural molecules that are present in the blood. A similar scenario was observed with α- and β-glycosidases, and in these cases, it was suggested that the synthetic substrates (like *p*-nitrophenyl or methyl-umbelliferyl glycosides) were analogs of glycoconjugates as glycolipids or glycoproteins. In this respect, even though it is clear that the AMC α-L-fucosidase was produced during the digestion of the blood meal in *R. prolixus*, it is not clear which kind of molecule it recognizes from the blood as a true digestive enzyme, or if this enzyme has an indirect role in the gut metabolism. More studies are necessary to elucidate the role of this enzyme, and especially its relation to the digestion of blood or interaction with microorganisms, especially because these roles may have a profound impact on the vectorial competence of *R. prolixus* for the transmission of *T. cruzi*.

## Data Availability

The original contributions presented in the study are included in the article/Supplementary Material, further inquiries can be directed to the corresponding author.

## Footnotes

1 PAHO. (2020). General Information - Chagas Disease. https://www.paho.org/hq/index.php?option=com_content&view=article&id=5856:2011-informacion-general-enfermedad-chagas&Itemid=40370&lang=en [Accessed 12 May 2020].

2 These gut compartments were also named by other authors as crop and intestine, or stomach and small intestine, respectively. We used anterior and posterior midgut to keep the nomenclature similar to what is used for other hemipteran groups.

3 Formerly known as *Nocardia rhodnii*.

## Supplementary material

SUPPLEMENTARY FIGURE S1 Stability of α-L-fucosidase from different *Rhodnius prolixus* gut fractions after 0–3 cycles of freezing and thawing. Total activities (µU/insect) were determined in independent samples that were submitted to 0 (no treatment, fresh samples), 1, 2 or 3 cycles of freezing and thawing. SG: salivary glands; AMC: anterior midgut contents; AMT: anterior midgut tissues; PMC: posterior midgut contents; PMT: posterior midgut tissues; HG: hindguts. N = 3 independent replicates. See Supplementary Table S1 for statistical analysis.

SUPPLEMENTARY FIGURE S2
*Rhodnius prolixus* weight and α-L-fucosidase activities before and after feeding with defibrinated blood (Control) or different blood fractions (Plasma; Cell fraction). **(A)** Weights of individual insects before and immediately after feeding. BF: Before feeding (black circles), AF: After feeding (gray squares). N = 20 insects in each group/condition. **(B)** α-L-fucosidase activity in different midgut fractions before feeding (BF) or 5 days after feeding. AMC: anterior midgut contents; AMT: anterior midgut tissues; PMC: posterior midgut contents; PMT: posterior midgut tissues. BF: before feeding corresponds to insects that were starved for 40 days. The groups marked with different letters (a,b,c) correspond to sets of values that are statistically different from each other (see Supplementary Table S2). n = 2 pools of 5 insects each. Longitudinal bars show the arithmetic mean of each data group.

SUPPLEMENTARY FIGURE S3 Protein concentrations (µg/µL of sample) in *Rhodnius prolixus* midgut fractions before and after feeding with defibrinated blood (Control) or different blood fractions. **(A)** Protein concentrations in Anterior Midgut Contents (AMC) and Anterior Midgut Tissues (AMT). **(B)** Protein concentrations in Posterior Midgut Contents (PMC) and Posterior Midgut Tissues (PMT). BF: Before Feeding corresponds to insects that were starved for 40 days. The columns marked with different letters (a, b, c) correspond to sets of values that are statistically different from each other (see Supplementary Table S3). N = 2 pools of 5 insects each. Error bars correspond to the Standard Deviation (SD) in each group.

SUPPLEMENTARY FIGURE S4 Protein concentrations (µg/µL of sample) in *Rhodnius prolixus* midgut fractions before and after feeding with citrated blood (Control) or different blood fractions. **(A)** Protein concentrations in Anterior Midgut Contents (AMC) and Anterior Midgut Tissues (AMT). **(B)** Protein concentrations in Posterior Midgut Contents (PMC) and Posterior Midgut Tissues (PMT). BF: before feeding corresponds to insects that were starved for 40 days. The columns marked with different letters (a, b, c) correspond to sets of values that are statistically different from each other (see Supplementary Table S5). N = 4 pools of 5 insects each. Error bars correspond to the Standard Deviation (SD) in each group.

SUPPLEMENTARY FIGURE S5 Protein concentrations (µg/µL of sample) in *Rhodnius prolixus* midgut fractions before and after feeding with heparinized blood (Control) or different blood fractions. **(A)** Protein concentrations in Anterior Midgut Contents (AMC) and Anterior Midgut Tissues (AMT). **(B)** Protein concentrations in Posterior Midgut Contents (PMC) and Posterior Midgut Tissues (PMT). BF: before feeding corresponds to insects that were starved for 40 days. PBS: Phosphate buffered saline (pH 7.2). The columns marked with different letters (a, b, c) correspond to sets of values that are statistically different from each other (see Supplementary Table S7). N = 6 pools of 5 insects each. Error bars correspond to the Standard Deviation (SD) in each group.

SUPPLEMENTARY FIGURE S6 Protein concentrations (µg/µL of sample) in *Rhodnius prolixus* AMC before and after feeding with heparinized blood (Control), different blood fractions after washing, and solutions of different proteins or sugars. BF: before feeding corresponds to insects that were starved for 40 days. PBS: Phosphate buffered saline (pH 7.2). HH: Human Hemoglobin; BH: Bovine Hemoglobin; RA: Rabbit Albumin, BA: Bovine Albumin. N = 6 pools of 5 insects each. The columns marked with different letters (a, b, c) correspond to sets of values that are statistically different from each other (see Supplementary Table S9). Error bars correspond to the Standard Deviation (SD) in each group.
