# Supplementary figures and images for: Digestive α-L-fucosidase activity in Rhodnius prolixus after blood feeding: effect of secretagogue and nutritional stimuli
Source: Front Physiol. 2023 Jul 19;14:1123414. doi: 10.3389/fphys.2023.1123414 (PMC10394381; doi:10.3389/fphys.2023.1123414)

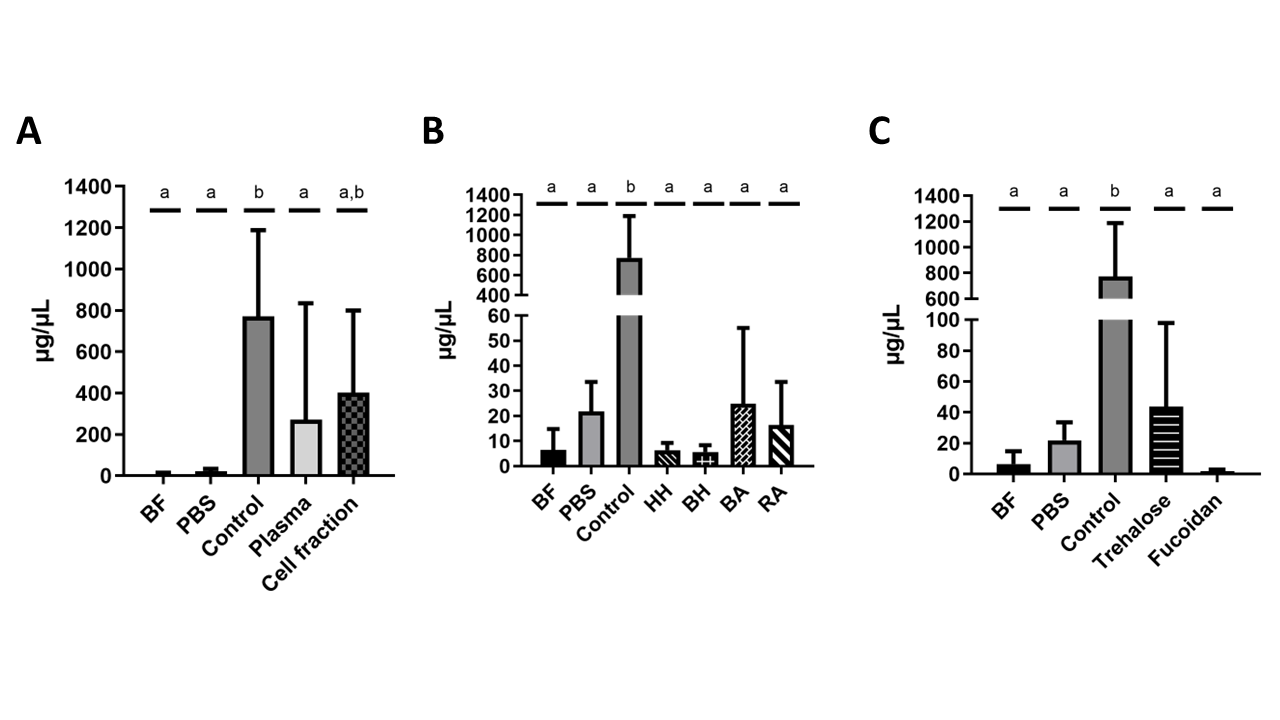

Supplement: Supplementary file 1 [file Image6.TIF]

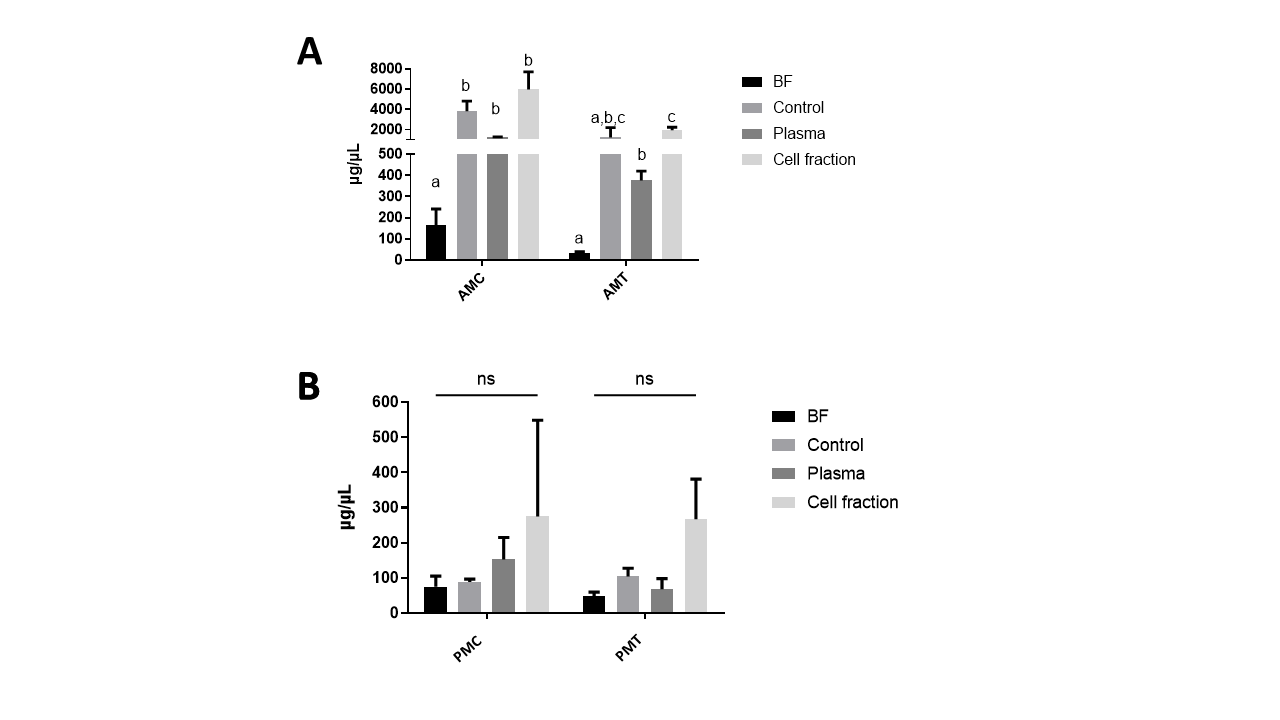

Supplement: Supplementary file 4 [file Image3.tif]

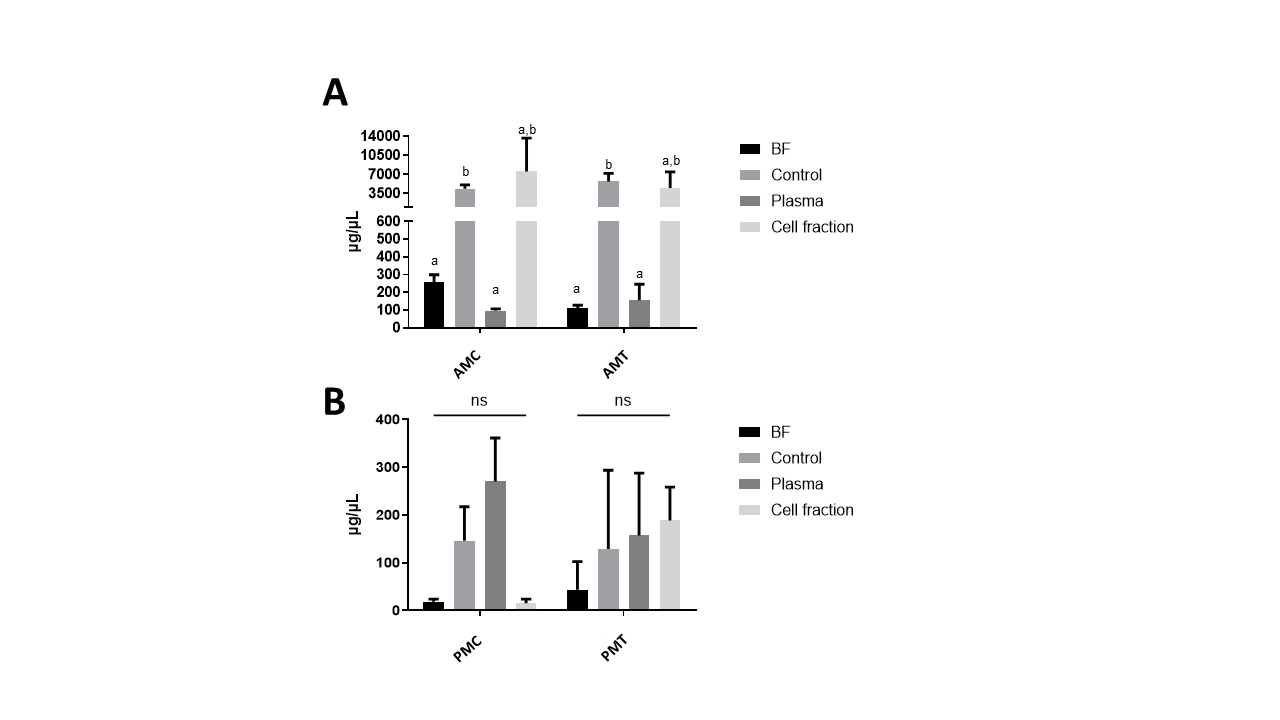

Supplement: Supplementary file 5 [file Image4.tif]

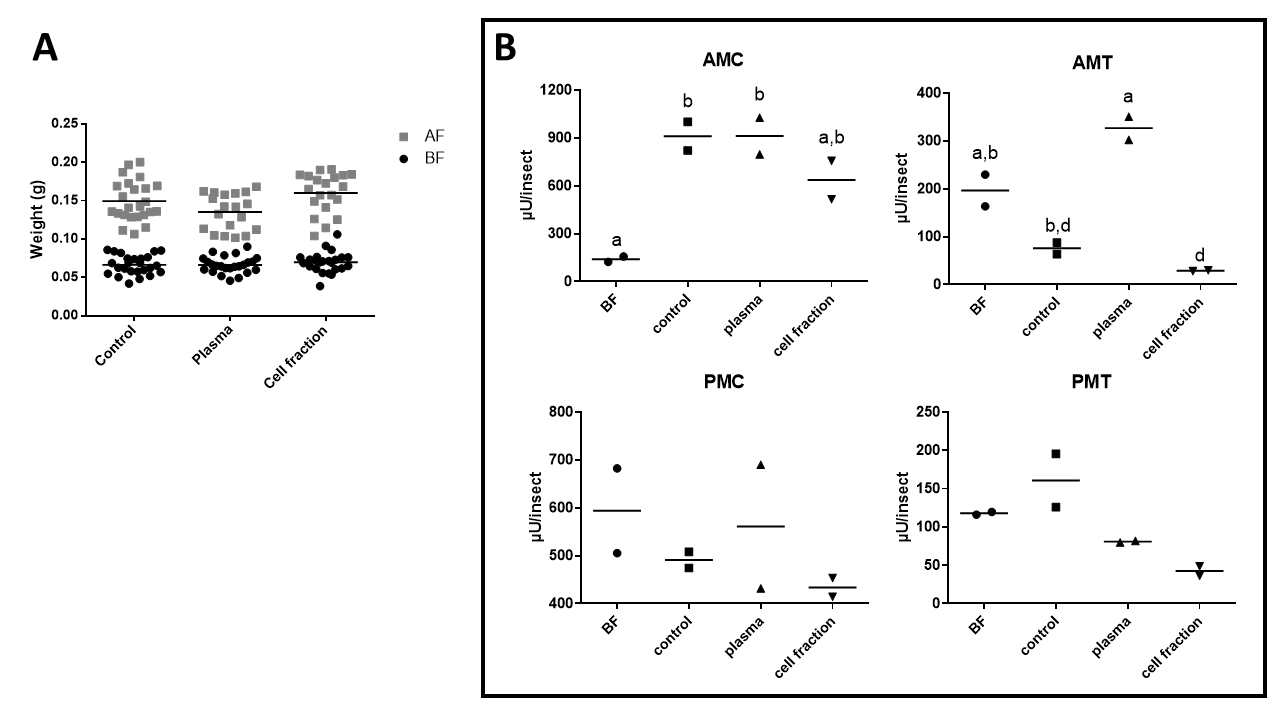

Supplement: Supplementary file 6 [file Image2.TIF]

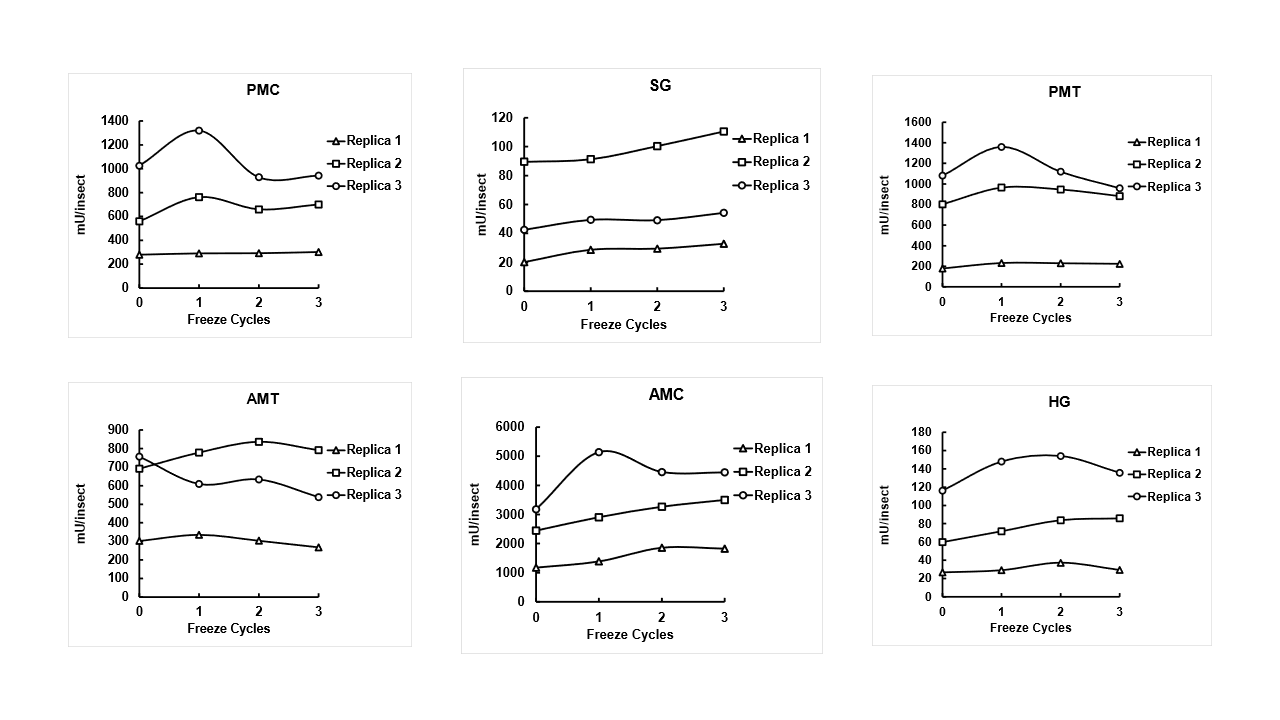

Supplement: Supplementary file 8 [file Image1.TIF]

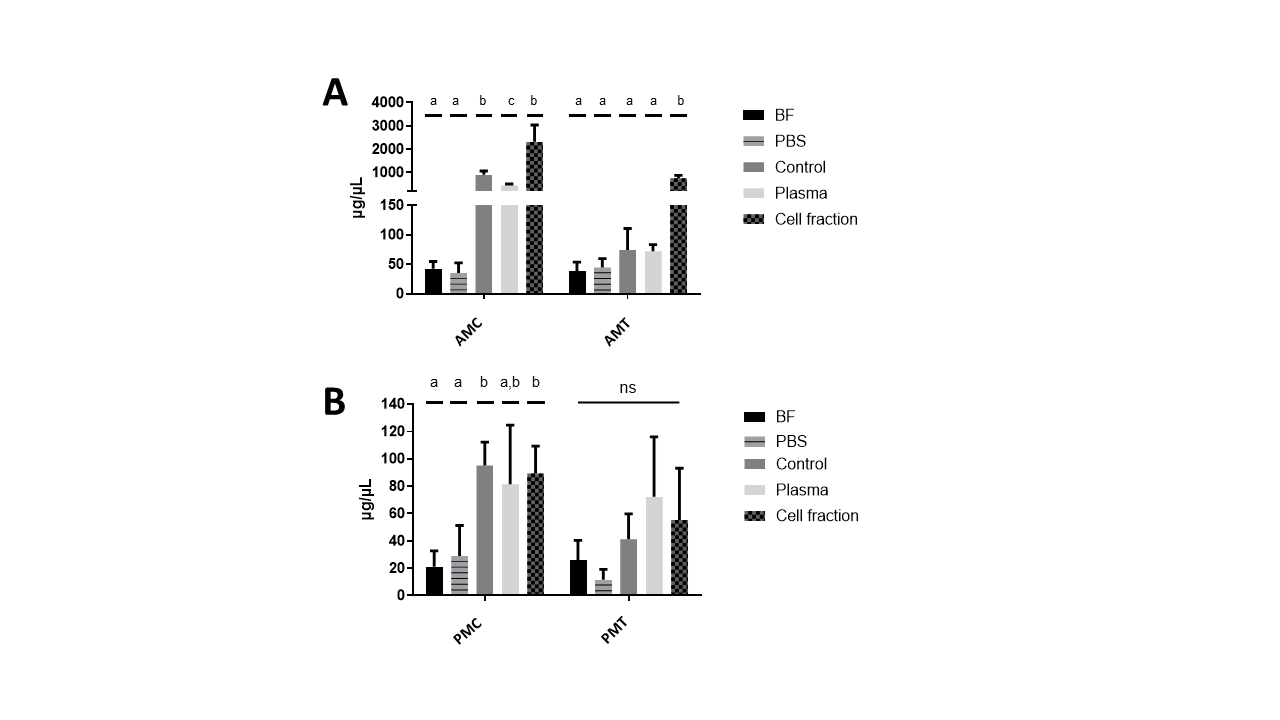

Supplement: Supplementary file 16 [file Image5.tif]
