# Supplementary material for: Digestive α-L-fucosidase activity in Rhodnius prolixus after blood feeding: effect of secretagogue and nutritional stimuli
Source: Front Physiol. 2023 Jul 19;14:1123414. doi: 10.3389/fphys.2023.1123414 (PMC10394381; doi:10.3389/fphys.2023.1123414)
Supplement: Supplementary file 2 [file Table1.docx]

Supplementary Table 1. Summary of statistical analysis of tha data from Supplementary Figure 1. Sign test was calculated comparing fresh samples with samples that suffered at least one cycle of freeze and thaw (1, 2 or 3). Spearman Correlation test was performed ranking the activity of samples according to the number of freeze/thaw cycles (0, 1, 2 or 3). N = 12 for all analysis.

| Sample | Sign Test | Spearman Correlation Test |
| --- | --- | --- |
| Salivary Glands | Z = 3 , ***p* = 0.0027** | r = 0.302 , T = 1.003, *p* = 0.340 |
| Anterior Midgut Contents | Z = 3 , ***p* = 0.0027** | r = 0.324 , T = 1.083 , *p* = 0.304 |
| Anterior Midgut Tissue | Z = 0.333 , *p* = 0.738 | r = -0.043 , T = 0.137 , *p* = 0.894 |
| Posterior Midgut Contents | Z = 1.667 , *p* = 0.096 | r = 0.086 , T = 0.274 , *p* = 0.79 |
| Posterior Midgut Tissue | Z = 2.333 , ***p* = 0.0196** | r = -0.043 , T = -0.137 , *p* = 0.984 |
| Hindgut | Z = 3 , ***p* = 0.0027** | r = 0.237 , T = 0.773 , *p* = 0.457 |
