# Supplementary material for: Digestive α-L-fucosidase activity in Rhodnius prolixus after blood feeding: effect of secretagogue and nutritional stimuli
Source: Front Physiol. 2023 Jul 19;14:1123414. doi: 10.3389/fphys.2023.1123414 (PMC10394381; doi:10.3389/fphys.2023.1123414)
Supplement: Supplementary file 3 [file Table8.docx]

Supplementary Table 8. Summary of the statistical analysis of data presented in Figure 3. (A) Comparisons of weights of insects before and after feeding with heparinated blood (Control), PBS, plasma and washed cells fractions. (B) Comparion of α-fucosidase activities in AMC samples obtained from insects before and after feeding with PBS, heparinated blood, plasma and washed cells fractions. BF – Before Feeding, AMC -Anterior Midgut Contents. (C) Comparison of refusal rates in groups of insects that were offered the different diets above (Fisher´s exact test). (D) Comparion of mortality 5 days after feeding with the different diets above (Fisher´s exact test).

(A)

| Sample Subset | Type of test | Comparison | Results | |
| --- | --- | --- | --- | --- |
| PBS | Unpaired T test | Before x After feeding | t (27) = 12.59 | ***p* < 0.0001** |
| Control | Unpaired T test | Before x After feeding | t (22) = 11.49 | ***p* < 0.0001** |
| Plasma | Unpaired T test | Before x After feeding | t (26) = 13.23 | ***p* < 0.0001** |
| Cell fraction | Unpaired T test | Before x After feeding | t (26) = 13.46 | ***p* < 0.0001** |
| Before feeding | One way ANOVA | Control x PBS x Plasma x Cell fraction | F (3, 39) = 2.039 | *p* = 0.1243 |
| After feeding | One way ANOVA | Control x PBS x Plasma x Cell fraction | F (3, 62) = 6.065 | ***p* = 0.0011** |
| After feeding | Tukey´s post hoc | Control x PBS | ***p* = 0.0321** |  |
| After feeding | Tukey´s post hoc | Control x Plasma | ***p* = 0.0031** |  |
| After feeding | Tukey´s post hoc | Control x Cell Fraction | ***p* = 0.0016** |  |
| After feeding | Tukey´s post hoc | PBS x Plasma | *p* = 0.7271 |  |
| After feeding | Tukey´s post hoc | PBS x Cell Fraction | *p* = 0.6678 |  |
| After feeding | Tukey´s post hoc | Plasma x Cell Fraction | *p* > 0.9999 |  |

(B)

| Sample Subset | Type of test | Comparison | Results | |
| --- | --- | --- | --- | --- |
| AMC | ANOVA | All groups | F (4, 74) = 25.12 | *p* < 0.0001 |
| AMC | Tukey´s post hoc | BF x PBS | *p* = 0.9892 | 95% C.I. = -1305 to 924.7 |
| AMC | Tukey´s post hoc | BF x Control | *p* < 0.0001 | 95% C.I. = -2823 to -750.0 |
| AMC | Tukey´s post hoc | BF x Plasma | *p* < 0.0001 | 95% C.I. = -4731 to -2457 |
| AMC | Tukey´s post hoc | BF x Cell Fraction | *p* = 0.0186 | 95% C.I. = -2236 to -138.2 |
| AMC | Tukey´s post hoc | PBS x Control | *p* = 0.0006 | 95% C.I. = -2653 to -539.2 |
| AMC | Tukey´s post hoc | PBS x Plasma | *p* < 0.0001 | 95% C.I. = -4560 to -2248 |
| AMC | Tukey´s post hoc | PBS x Cell Fraction | *p* = 0.0794 | 95% C.I. = -2066 to 72.38 |
| AMC | Tukey´s post hoc | Control x Plasma | *p* = 0.0001 | 95% C.I. = -2888 to -727.8 |
| AMC | Tukey´s post hoc | Control x Cell Fraction | *p* = 0.4418 | 95% C.I. = -387.8 to 1586 |
| AMC | Tukey´s post hoc | Plasma x Cell Fraction | *p* < 0.0001 | 95% C.I. = 1315 to 3499 |

(C)

|  | Group 1 | | Group 2 | |  |
| --- | --- | --- | --- | --- | --- |
| Comparison | N Fed | N Refused | N Fed | N Refused | two-tailed *p* |
| PBS x Control | 26 | 3 | 24 | 1 | 0.6149 |
| PBS x Plasma | 26 | 3 | 22 | 5 | 0.4620 |
| PBS x Cell Fraction | 26 | 3 | 23 | 3 | 1.0000 |
| Control x Plasma | 24 | 1 | 22 | 5 | 0.1928 |
| Control x Cell Fraction | 24 | 1 | 23 | 3 | 0.6098 |
| Plasma x Cell Fraction | 22 | 5 | 23 | 3 | 0.7040 |

(D)

|  | Group 1 | | Group 2 | |  |
| --- | --- | --- | --- | --- | --- |
| Comparison | N Live | N Dead | N Live | N Dead | two-tailed *p* |
| PBS x Control | 15 | 1 | 19 | 3 | 0.6245 |
| PBS x Plasma | 15 | 1 | 19 | 3 | 0.6245 |
| PBS x Cell Fraction | 15 | 1 | 22 | 2 | 1.0000 |
| Control x Plasma | 19 | 3 | 19 | 3 | 1.0000 |
| Control x Cell Fraction | 19 | 3 | 22 | 2 | 0.6589 |
| Plasma x Cell Fraction | 19 | 3 | 22 | 2 | 0.6589 |
