# Supplementary material for: Digestive α-L-fucosidase activity in Rhodnius prolixus after blood feeding: effect of secretagogue and nutritional stimuli
Source: Front Physiol. 2023 Jul 19;14:1123414. doi: 10.3389/fphys.2023.1123414 (PMC10394381; doi:10.3389/fphys.2023.1123414)
Supplement: Supplementary file 7 [file Table7.docx]

Supplementary Table 7. Summary of the statistical analysis of data presented in Supplementary Figure 5. Comparisons of protein concentrations between midgut samples obtained from insects before and after feeding with heparinated blood (Control), PBS, plasma and cell fractions. BF – Before Feeding, AMC - Anterior Midgut Contents, AMT - Anterior Midgut Tissue, PMC - Posterior Midgur Contents, and PMT - Posterior Midgut Tissue (PMT).

| Sample Subset | Type of test | Comparison | Results | | Statistical Power |
| --- | --- | --- | --- | --- | --- |
| AMC | Unpaired T test | BF x PBS | (M = 42.74, SD = 12.21)  (M = 35.38, SD = 17.29) | t (5) = 0.6663  *p* = 0.5347 | 0.192 |
| AMC | Unpaired T test | BF x Control | (M = 42.74, SD = 12.21)  (M = 904.7, SD = 161.1) | t (4) = 12.26  ***p* = 0.0003** | **1** |
| AMC | Unpaired T test | BF x Plasma | (M = 42.74, SD = 12.21)  (M = 435.8, SD = 68.93) | t (6) = 11.23  ***p* < 0.0001** | **1** |
| AMC | Unpaired T test | BF x Cell Fraction | (M = 42.74, SD = 12.21)  (M = 2317, SD = 719.7) | t (6) = 6.320  ***p* = 0.0007** | **1** |
| AMC | Unpaired T test | PBS x Control | (M = 35.38, SD = 17.29)  (M = 904.7, SD = 161.1) | t (3) = 10.13  ***p* = 0.0021** | **1** |
| AMC | Unpaired T test | PBS x Plasma | (M = 35.38, SD = 17.29)  (M = 435.8, SD = 68.93) | t (5) = 9.620  ***p* = 0.0002** | **1** |
| AMC | Unpaired T test | PBS x Cell Fraction | (M = 35.38, SD = 17.29)  (M = 2317, SD = 719.7) | t (5) = 5.358  ***p* = 0.0030** | **1** |
| AMC | Unpaired T test | Control x Plasma | (M = 904.7, SD = 161.1)  (M = 435.8, SD = 68.93) | t (4) = 5.402  ***p* = 0.0057** | 0.0859 |
| AMC | Unpaired T test | Control x Cell Fraction | (M = 904.7, SD = 161.1)  (M = 2317, SD = 719.7) | t (4) = 2.595  *p* = 0.0604 | 0.84 |
| AMC | Unpaired T test | Plasma x Cell Fraction | (M = 435.8, SD = 68.93)  (M = 2317, SD = 719.7) | t (6) = 5.204  ***p* = 0.0020** | **1** |
| AMT | Unpaired T test | BF x PBS | (M = 38.10, SD = 15.80)  (M = 44.71, SD = 15.20) | t (6) = 0.6034  *p* = 0.5683 | 0.38 |
| AMT | Unpaired T test | BF x Control | (M = 38.10, SD = 15.80)  (M = 74.57, SD = 36.26) | t (5) = 1.837  *p* = 0.1256 | 0.728 |
| AMT | Unpaired T test | BF x Plasma | (M = 38.10, SD = 15.80)  (M = 72.31, SD = 11.42) | t (4) = 2.665  *p* = 0.0561 | 0.593 |
| AMT | Unpaired T test | BF x Cell Fraction | (M = 38.10, SD = 15.80)  (M = 754.1, SD =119;4) | t (5) = 12.26  ***p* < 0.001** | **1** |
| AMT | Unpaired T test | PBS x Control | (M = 44.71, SD = 15.20)  (M = 74.57, SD = 36.26) | t (5) = 1.517  *p* = 0.1898 | 0.59 |
| AMT | Unpaired T test | PBS x Plasma | (M = 44.71, SD = 15.20)  (M = 72.31, SD = 11.42) | t (4) = 2.222  *p* = 0.0905 | 0.464 |
| AMT | Unpaired T test | PBS x Cell Fraction | (M = 44.71, SD = 15.20)  (M = 754.1, SD = 119.4) | t (5) = 12.15  ***p* < 0.0001** | **1** |
| AMT | Unpaired T test | Control x Plasma | (M = 74.57, SD = 36.26)  (M = 72.31, SD = 11.42) | t (3) = 0.08172  *p* = 0.9400 | 0.186 |
| AMT | Unpaired T test | Control x Cell Fraction | (M = 74.57, SD = 36.26)  (M = 754.1, SD = 119.4) | t (4) = 9.433  ***p* = 0.0007** | **1** |
| AMT | Unpaired T test | Plasma x Cell Fraction | (M = 72.31, SD = 11.42)  (M = 754.1, SD = 119.4) | t (3) = 7.644  ***p* = 0.0046** | **1** |
| PMC | Unpaired T test | BF x PBS | (M = 21.07, SD = 11.70)  (M = 28.91, SD = 22.40) | t (6) = 0.6204  *p* = 0.5578 | 0.491 |
| PMC | Unpaired T test | BF x Control | (M = 21.07, SD = 11.70)  (M = 95.14, SD = 17.21) | t (5) = 6.847  ***p* = 0.0010** | **1** |
| PMC | Unpaired T test | BF x Plasma | (M = 21.07, SD = 11.70)  (M = 81.33, SD = 43.46) | t (5) = 2.727  ***p* = 0.0415** | **0.999** |
| PMC | Unpaired T test | BF x Cell Fraction | (M = 21.07, SD = 11.70)  (M = 91.57, SD = 16.93) | t (4) = 6.166  ***p* = 0.0035** | **0.999** |
| PMC | Unpaired T test | PBS x Control | (M = 28.91, SD = 22.40)  (M = 95.14, SD = 17.21) | t (5) = 4.234  ***p* = 0.0082** | **0.991** |
| PMC | Unpaired T test | PBS x Plasma | (M = 28.91, SD = 22.40)  (M = 81.33, SD = 43.46) | t (5) = 2.112  *p* = 0.0884 | 0.957 |
| PMC | Unpaired T test | PBS x Cell Fraction | (M = 28.91, SD = 22.40)  (M = 91.57, SD = 16.93) | t (4) = 3.419  ***p* = 0.0268** | **0.955** |
| PMC | Unpaired T test | Control x Plasma | (M = 95.14, SD = 17.21)  (M = 81.33, SD = 43.46) | t (4) = 0.5117  *p* = 0.6358 | 0.18 |
| PMC | Unpaired T test | Control x Cell Fraction | (M = 95.14, SD = 17.21)  (M = 91.57, SD = 16.93) | t (3) = 0.2285  *p* = 0.8339 | 0.183 |
| PMC | Unpaired T test | Plasma x Cell Fraction | (M = 81.33, SD = 43.46)  (M = 91.57, SD = 16.93) | t (3) = 0.3047  *p* = 0.7805 | 0.236 |
| PMT | Unpaired T test | BF x PBS | (M = 25.90, SD = 14.48)  (M = 11.66, SD = 7.495) | t (5) = 1.531  *p* = 0.1864 | 0.0906 |
| PMT | Unpaired T test | BF x Control | (M = 25.90, SD = 14.48)  (M = 41.29, SD = 18.47) | t (5) = 1.244  *p* = 0.2686 | 0.549 |
| PMT | Unpaired T test | BF x Plasma | (M = 25.90, SD = 14.48)  (M = 72.25, SD = 43.84) | t (6) = 2.008  *p* = 0.0914 | 0.976 |
| PMT | Unpaired T test | BF x Cell Fraction | (M = 25.90, SD = 14.48)  (M = 55.37, SD = 37.73) | t (5) = 1.464  *p* = 0.2032 | 0.8 |
| PMT | Unpaired T test | PBS x Control | (M = 11.66, SD = 7.495)  (M = 41.29, SD = 18.47) | t (4) = 2.574  *p* = 0.0617 | 0.991 |
| PMT | Unpaired T test | PBS x Plasma | (M = 11.66, SD = 7.495)  (M = 72.25, SD = 43.84) | t (5) = 2.314  *p* = 0.0686 | 1 |
| PMT | Unpaired T test | PBS x Cell Fraction | (M = 11.66, SD = 7.495)  (M = 55.37, SD = 37.73) | t (4) = 1.968  *p* = 0.1204 | 1 |
| PMT | Unpaired T test | Control x Plasma | (M = 41.29, SD = 18.47)  (M = 72.25, SD = 43.84) | t (5) = 1.129  *p* = 0.3101 | 0.63 |
| PMT | Unpaired T test | Control x Cell Fraction | (M = 41.29, SD = 18.47)  (M = 55.37, SD = 37.73) | t (4) = 0.5808  *p* = 0.5925 | 0.375 |
| PMT | Unpaired T test | Plasma x Cell Fraction | (M = 72.25, SD = 43.84)  (M = 55.37, SD = 37.73) | t (5) = 0.5324  *p* = 0.6172 | 0.172 |
