# Supplementary material for: Digestive α-L-fucosidase activity in Rhodnius prolixus after blood feeding: effect of secretagogue and nutritional stimuli
Source: Front Physiol. 2023 Jul 19;14:1123414. doi: 10.3389/fphys.2023.1123414 (PMC10394381; doi:10.3389/fphys.2023.1123414)
Supplement: Supplementary file 9 [file Table9.docx]

Supplementary Table 9. Summary of the statistical analysis of data presented in Supplementary Figure 6. (A) Comparisons of protein concentrations between AMC samples obtained from insects before and after feeding with , PBS, heparinated blood (Control), plasma and washed cells fraction (data in Supplemental Figure 6A) (B) Comparisons of protein concentrations between AMC samples obtained from insects before and after feeding with PBS, heparinated blood (Control), Human Hemoglobin (HH), Bovine Hemoglobin (BH), Bovine Albumin (BA), and Rabbit Albumin (RA) (data in Supplemental Figure 6B. (C) Comparisons of protein concentrations between AMC samples obtained from insects before and after feeding with PBS, heparinated blood (Control), trehalose and fucose. BF – Before Feeding, AMC - Anterior Midgut Contents.

(A)

| Sample Subset | Type of test | Comparison | Results | |
| --- | --- | --- | --- | --- |
| AMC | ANOVA | All groups | F (4, 50) = 7.494 | *p* < 0.0001 |
| AMC | Tukey´s post hoc | BF x PBS | *p* > 0.9999 | 95% C.I. = -455.2 to 424.5 |
| AMC | Tukey´s post hoc | BF x Control | *p* < 0.0001 | 95% C.I. = -1205 to -325.6 |
| AMC | Tukey´s post hoc | BF x Plasma | *p* = 0.3351 | 95% C.I. = -661.6 to 131.5 |
| AMC | Tukey´s post hoc | BF x Cell Fraction | *p* = 0.0809 | 95% C.I. = -821.7 to 30.81 |
| AMC | Tukey´s post hoc | PBS x Control | *p* = 0.0006 | 95% C.I. = -1235 to -264.8 |
| AMC | Tukey´s post hoc | PBS x Plasma | *p* = 0.5149 | 95% C.I. = -696.2 to 196.7 |
| AMC | Tukey´s post hoc | PBS x Cell Fraction | *p* = 0.1705 | 95% C.I. = -853.2 to 92.90 |
| AMC | Tukey´s post hoc | Control x Plasma | *p* = 0.0209 | 95% C.I. = 53.90 to 946.8 |
| AMC | Tukey´s post hoc | Control x Cell Fraction | *p* = 0.1917 | 95% C.I. = -103.1 to 843.0 |
| AMC | Tukey´s post hoc | Plasma x Cell Fraction | *p* = 0.9127 | 95% C.I. = -563.4 to 302.7 |

(B)

| Sample Subset | Type of test | Comparison | Results | |
| --- | --- | --- | --- | --- |
| AMC | ANOVA | All groups | F (6, 61) = 32.69 | *p* < 0.0001 |
| AMC | Tukey´s post hoc | BF x PBS | *p* > 0.9999 | 95% C.I. = -212.7 to 182.1 |
| AMC | Tukey´s post hoc | BF x Control | ***p* < 0.0001** | 95% C.I. = -962.8 to -568.0 |
| AMC | Tukey´s post hoc | BF x HH | *p* > 0.9999 | 95% C.I. = -204.7 to 204.9 |
| AMC | Tukey´s post hoc | BF x BH | *p* > 0.9999 | 95% C.I. = -185.3 to 187.0 |
| AMC | Tukey´s post hoc | BF x BA | *p* > 0.9999 | 95% C.I. = -215.8 to 179.0 |
| AMC | Tukey´s post hoc | BF x RA | *p* > 0.9999 | 95% C.I. = -214.6 to 194.9 |
| AMC | Tukey´s post hoc | PBS x Control | ***p* < 0.0001** | 95% C.I. = -967.9 to -532.3 |
| AMC | Tukey´s post hoc | PBS x HH | *p* > 0.9999 | 95% C.I. = -209.1 to 239.9 |
| AMC | Tukey´s post hoc | PBS x BH | *p* > 0.9999 | 95% C.I. = -191.5 to 223.8 |
| AMC | Tukey´s post hoc | PBS x BA | *p* > 0.9999 | 95% C.I. = -220.9 to 214.7 |
| AMC | Tukey´s post hoc | PBS x RA | *p* > 0.9999 | 95% C.I. = -219.0 to 230.0 |
| AMC | Tukey´s post hoc | Control x HH | ***p* < 0.0001** | 95% C.I. = 541.0 to 990.0 |
| AMC | Tukey´s post hoc | Control x BH | ***p* < 0.0001** | 95% C.I. = 558.6 to 973.9 |
| AMC | Tukey´s post hoc | Control x BA | ***p* < 0.0001** | 95% C.I. = 529.2 to 964.8 |
| AMC | Tukey´s post hoc | Control x RA | ***p* < 0.0001** | 95% C.I. = 531.1 to 980.1 |
| AMC | Tukey´s post hoc | HH x BH | *p* > 0.9999 | 95% C.I. = -213.9 to 215.5 |
| AMC | Tukey´s post hoc | HH x BA | *p* > 0.9999 | 95% C.I. = -243.0 to 206.0 |
| AMC | Tukey´s post hoc | HH x RA | *p* > 0.9999 | 95% C.I. = -241.0 to 221.0 |
| AMC | Tukey´s post hoc | BH x BA | *p* > 0.9999 | 95% C.I. = -226.9 to 188.4 |
| AMC | Tukey´s post hoc | BH x RA | *p* > 0.9999 | 95% C.I. = -225.4 to 203.9 |
| AMC | Tukey´s post hoc | BA x RA | *p* > 0.9999 | 95% C.I. = -215.9 to 233.0 |

(C)

| Sample Subset | Type of test | Comparison | Results | |
| --- | --- | --- | --- | --- |
| AMC | ANOVA | All groups | F (4, 39) = 27.84 | ***p* < 0.0001** |
| AMC | Tukey´s post hoc | BF x PBS | *p* = 0.9997 | 95% C.I. = -248.3 to 217.6 |
| AMC | Tukey´s post hoc | BF x Control | ***p* < 0.0001** | 95% C.I. = -998.4 to -532.5 |
| AMC | Tukey´s post hoc | BF x Trehalose | *p* = 0.9896 | 95% C.I. = -262.9 to 188.6 |
| AMC | Tukey´s post hoc | BF x Fucoidan | *p* > 0.9999 | 95% C.I. = -407.7 to 416.5 |
| AMC | Tukey´s post hoc | PBS x Control | ***p* < 0.0001** | 95% C.I. = -1007 to -493.1 |
| AMC | Tukey´s post hoc | PBS x Trehalose | *p* = 0.9991 | 95% C.I. = -272.3 to 228.7 |
| AMC | Tukey´s post hoc | PBS x Fucoidan | *p* > 0.9999 | 95% C.I. = -406.5 to 445.9 |
| AMC | Tukey´s post hoc | Control x Trehalose | *p* < 0.0001 | 95% C.I. = 477.8 to 978.8 |
| AMC | Tukey´s post hoc | Control x Fucoidan | *p* < 0.0001 | 95% C.I. = 343.6 to 1196 |
| AMC | Tukey´s post hoc | Trehalose x Fucoidan | *p* = 0.9986 | 95% C.I. = -380.8 to 463.9 |
