# Supplementary material for: Digestive α-L-fucosidase activity in Rhodnius prolixus after blood feeding: effect of secretagogue and nutritional stimuli
Source: Front Physiol. 2023 Jul 19;14:1123414. doi: 10.3389/fphys.2023.1123414 (PMC10394381; doi:10.3389/fphys.2023.1123414)
Supplement: Supplementary file 11 [file Table5.docx]

Supplementary Table 5. Summary of the statistical analysis of data presented in Supplementary Figure 4. Comparisons of protein concentrations between midgut samples obtained from insects before and after feeding with citrated blood (Control), plasma and cell fractions. BF – Before Feeding, AMC - Anterior Midgut Contents, AMT - Anterior Midgut Tissue, PMC - Posterior Midgur Contents, and PMT - Posterior Midgut Tissue (PMT).

| Sample Subset | Type of test | Comparison | Results | | Statistical Power |
| --- | --- | --- | --- | --- | --- |
| AMC | Unpaired T test | BF x Control | (M = 256.6, SD = 43.26)  (M = 4379 SD = 695.34) | t (2) = 8.367  ***p* = 0.0140** | **1** |
| AMC | Unpaired T test | BF x Plasma | (M = 256.6, SD = 43.26)  (M = 95.51 SD = 11.17) | t (2) = 5.101  *p* = 0.0364 | 0.767 |
| AMC | Unpaired T test | BF x Cell Fraction | (M = 256.6, SD = 43.26)  (M = 7557 SD = 6102.00) | t (2) = 1.692  *p* = 0.2328 | 1 |
| AMC | Unpaired T test | Control x Plasma | (M = 4379 SD = 695.34)  (M = 95.51 SD = 11.17) | t (2) = 8.710  ***p* = 0.0129** | **1** |
| AMC | Unpaired T test | Control x Cell Fraction | (M = 4379 SD = 695.34)  (M = 7557 SD = 6102.00) | t (2) = 0.7318  *p* = 0.5404 | 0.409 |
| AMC | Unpaired T test | Plasma x Cell Fraction | (M = 95.51 SD = 11.17)  (M = 7557 SD = 6102.00) | t (2) = 1.729  *p* = 0.2259 | 1 |
| AMT | Unpaired T test | BF x Control | (M = 108.2 SD = 19.23)  (M = 5711 SD = 1470.99) | t (2) = 5.386  ***p* = 0.0328** | 1 |
| AMT | Unpaired T test | BF x Plasma | (M = 108.2 SD = 19.23)  (M = 155.8 SD = 90.20) | t (2) = 0.7307  *p* = 0.5410 | 0.302 |
| AMT | Unpaired T test | BF x Cell Fraction | (M = 108.2 SD = 19.23)  (M = 4505 SD = 2990.94) | t (2) = 2.079  *p* = 0.1732 | 1 |
| AMT | Unpaired T test | Control x Plasma | (M = 5711 SD = 1470.99)  (M = 155.8 SD = 90.20) | t (2) = 5.331  ***p* = 0.0334** | 1 |
| AMT | Unpaired T test | Control x Cell Fraction | (M = 5711 SD = 1470.99)  (M = 4505 SD = 2990.94) | t (2) = 0.5117  *p* = 0.6597 | 0.246 |
| AMT | Unpaired T test | Plasma x Cell Fraction | (M = 155.8 SD = 90.20)  (M = 4505 SD = 2990.94) | t (2) = 2.055  *p* = 0.1762 | 1 |
| PMC | Unpaired T test | BF x Control | (M = 18.99 SD = 5.33)  (M = 146.3 SD = 71.30) | t (2) = 2.518  *p* = 0.1281 | 1 |
| PMC | Unpaired T test | BF x Plasma | (M = 18.99 SD = 5.33)  (M = 270.5 SD = 90.94) | t (2) = 3.905  *p* = 0.0598 | 1 |
| PMC | Unpaired T test | BF x Cell Fraction | (M = 18.99 SD = 5.33)  (M = 15.53 SD = 8.56) | t (2) = 0.4849  *p* = 0.6757 | 0.23 |
| PMC | Unpaired T test | Control x Plasma | (M = 146.3 SD = 71.30)  (M = 270.5 SD = 90.94) | t (2) = 1.520  *p* = 0.2678 | 0.456 |
| PMC | Unpaired T test | Control x Cell Fraction | (M = 146.3 SD = 71.30)  (M = 15.53 SD = 8.56) | t (2) = 2.575  *p* = 0.1235 | 1 |
| PMC | Unpaired T test | Plasma x Cell Fraction | (M = 270.5 SD = 90.94)  (M = 15.53 SD = 8.56) | t (2) = 3.948  *p* = 0.0586 | 1 |
| PMT | Unpaired T test | BF x Control | (M = 43.99 SD = 58.49)  (M = 129.2 SD = 164.79) | t (2) = 0.6888  *p* = 0.5621 | **0.836** |
| PMT | Unpaired T test | BF x Plasma | (M = 43.99 SD = 58.49)  (M = 157.4 SD = 130.33) | t (2) = 1.123  *p* = 0.3781 | **0.947** |
| PMT | Unpaired T test | BF x Cell Fraction | (M = 43.99 SD = 58.49)  (M = 188.6 SD = 69.95) | t (2) = 2.243  *p* = 0.1541 | **0.990** |
| PMT | Unpaired T test | Control x Plasma | (M = 129.2 SD = 164.79)  (M = 157.4 SD = 130.33) | t (2) = 0.1904  *p* = 0.8666 | **0.230** |
| PMT | Unpaired T test | Control x Cell Fraction | (M = 129.2 SD = 164.79)  (M = 188.6 SD = 69.95) | t (2) = 0.4696  *p* = 0.6848 | **0.309** |
| PMT | Unpaired T test | Plasma x Cell Fraction | (M = 157.4 SD = 130.33)  (M = 188.6 SD = 69.95) | t (2) = 0.2980  *p* = 0.7938 | **0.224** |
