# Supplementary material for: Digestive α-L-fucosidase activity in Rhodnius prolixus after blood feeding: effect of secretagogue and nutritional stimuli
Source: Front Physiol. 2023 Jul 19;14:1123414. doi: 10.3389/fphys.2023.1123414 (PMC10394381; doi:10.3389/fphys.2023.1123414)
Supplement: Supplementary file 14 [file Table11.docx]

Supplementary Table 11. Summary of the statistical analysis of data presented in Figure 5. (A) Comparisons of weights of insects before and after feeding with PBS, heparinated blood (Control), Trehalose, Fucose, and Fucoidan. (B) Comparion of α-fucosidase activities of AMC samples prepared from insects before and after feeding with PBS, heparinated blood (Control), Trehalose, Fucose, and Fucoidan. BF – Before Feeding. (C) Comparison of refusal rates in groups of insects that were offered the different diets above (Fisher´s exact test). (D) Comparion of mortality 5 days after feeding with the different diets above (Fisher´s exact test).

(A)

| Sample Subset | Type of test | Comparison | Results | |
| --- | --- | --- | --- | --- |
| PBS | Unpaired T test | Before x After feeding | t (27) = 12.59 | ***p* < 0.0001** |
| Control | Unpaired T test | Before x After feeding | t (22) = 11.49 | ***p* < 0.0001** |
| Trehalose | Unpaired T test | Before x After feeding | t (38) = 12.40 | ***p* < 0.0001** |
| Fucose | Unpaired T test | Before x After feeding | t (23) = 7.835 | ***p* < 0.0001** |
| Fucoidan | Unpaired T test | Before x After feeding | t (23) = 12.06 | ***p* < 0.0001** |
| Before feeding | One way ANOVA | PBS x Control x Trehalose x Fucose x Fucoidan | F (4, 57) = 3.189 | ***p* = 0.0197** |
| Before feeding | Tukey´s post hoc | PBS x Control | *p* = 0.3317 | 95% C.I. = -0.003527 to 0.01803 |
| Before feeding | Tukey´s post hoc | PBS x Trehalose | *p* = 0.9661 | 95% C.I. = -0.01228 to 0.007679 |
| Before feeding | Tukey´s post hoc | PBS x Fucose | *p* = 0.8742 | 95% C.I. = -0.01381 to 0.006823 |
| Before feeding | Tukey´s post hoc | PBS x Fucoidan | *p* = 0.7726 | 95% C.I. = -0.01372 to 0.005706 |
| Before feeding | Tukey´s post hoc | Control x Trehalose | *p* = 0.0670 | 95% C.I. = -0.01953 to 0.0004287 |
| Before feeding | Tukey´s post hoc | Control x Fucose | ***p* = 0.0373** | 95% C.I. = -0.02106 to -0.0004271 |
| Before feeding | Tukey´s post hoc | Control x Fucoidan | ***p* = 0.0153** | 95% C.I. = -0.02097 to -0.001544 |
| Before feeding | Tukey´s post hoc | Trehalose x Fucose | *p* = 0.9965 | 95% C.I. = -0.01068 to 0.008283 |
| Before feeding | Tukey´s post hoc | Trehalose x Fucoidan | *p* = 0.9820 | 95% C.I. = -0.01053 to 0.007110 |
| Before feeding | Tukey´s post hoc | Fucose x Fucoidan | *p* = 0.9999 | 95% C.I. = -0.009715 to 0.008690 |
| After feeding | One way ANOVA | PBS x Control x Trehalose x Fucose x Fucoidan | F (4, 76) = 7.787 | ***p* < 0.0001** |
| After feeding | Tukey´s post hoc | PBS x Control | ***p* = 0.0333** | 95% C.I. = 0.001311 to 0.04830 |
| After feeding | Tukey´s post hoc | PBS x Trehalose | *p* = 0.3862 | 95% C.I. = -0.007228 to 0.03304 |
| After feeding | Tukey´s post hoc | PBS x Fucose | ***p* < 0.0001** | 95% C.I. = 0.02210 to 0.07012 |
| After feeding | Tukey´s post hoc | PBS x Fucoidan | *p* = 0.3177 | 95% C.I. = -0.008482 to 0.04551 |
| After feeding | Tukey´s post hoc | Control x Trehalose | *p* = 0.5632 | 95% C.I. = -0.03402 to 0.01021 |
| After feeding | Tukey´s post hoc | Control x Fucose | *p* = 0.1509 | 95% C.I. = -0.004392 to 0.04700 |
| After feeding | Tukey´s post hoc | Control x Fucoidan | *p* = 0.9719 | 95% C.I. = -0.03480 to 0.02221 |
| After feeding | Tukey´s post hoc | Trehalose x Fucose | ***p* = 0.0010** | 95% C.I. = 0.01054 to 0.05586 |
| After feeding | Tukey´s post hoc | Trehalose x Fucoidan | *p* = 0.9735 | 95% C.I. = -0.02019 to 0.03141 |
| After feeding | Tukey´s post hoc | Fucose x Fucoidan | *p* = 0.0687 | 95% C.I. = -0.05652 to 0.001330 |

(B)

| Sample Subset | Type of test | Comparison | Results | |
| --- | --- | --- | --- | --- |
| AMC | ANOVA | All groups | F (5, 63) = 11.14 | P<0.0001 |
| AMC | Tukey´s post hoc | BF x PBS | *p* = 0.9984 | 95% C.I. = -1543 to 1163 |
| AMC | Tukey´s post hoc | BF x Control | *p* = 0.0013 | 95% C.I. = -3044 to -529.0 |
| AMC | Tukey´s post hoc | BF x Trehalose | *p* = 0.6447 | 95% C.I. = -2425 to 762.6 |
| AMC | Tukey´s post hoc | BF x Fucose | *p* = 0.0162 | 95% C.I. = -3767 to -249.6 |
| AMC | Tukey´s post hoc | BF x Fucoidan | *p* < 0.0001 | 95% C.I. = -5200 to -1868 |
| AMC | Tukey´s post hoc | PBS x Control | *p* = 0.0066 | 95% C.I. = -2878 to -313.8 |
| AMC | Tukey´s post hoc | PBS x Trehalose | *p* = 0.8505 | 95% C.I. = -2254 to 972.7 |
| AMC | Tukey´s post hoc | PBS x Fucose | *p* = 0.0419 | 95% C.I. = -3594 to -41.43 |
| AMC | Tukey´s post hoc | PBS x Fucoidan | *p* < 0.0001 | 95% C.I. = -5029 to -1658 |
| AMC | Tukey´s post hoc | Control x Trehalose | *p* = 0.4545 | 95% C.I. = -579.1 to 2490 |
| AMC | Tukey´s post hoc | Control x Fucose | *p* = 0.9989 | 95% C.I. = -1927 to 1483 |
| AMC | Tukey´s post hoc | Control x Fucoidan | *p* = 0.0257 | 95% C.I. = -3357 to -137.9 |
| AMC | Tukey´s post hoc | Trehalose x Fucose | *p* = 0.4988 | 95% C.I. = -3143 to 789.2 |
| AMC | Tukey´s post hoc | Trehalose x Fucoidan | *p* = 0.0011 | 95% C.I. = -4587 to -818.6 |
| AMC | Tukey´s post hoc | Fucose x Fucoidan | *p* = 0.2460 | 95% C.I. = -3551 to 499.7 |

(C)

|  | Group 1 | | Group 2 | |  |
| --- | --- | --- | --- | --- | --- |
| Comparison | N Fed | N Refused | N Fed | N Refused | two-tailed *p* |
| PBS x Control | 26 | 3 | 24 | 1 | 0.6149 |
| PBS x Trehalose | 26 | 3 | 34 | 6 | 0.7242 |
| PBS x Fucose | 26 | 3 | 17 | 13 | 0.0074 |
| PBS x Fucoidan | 26 | 3 | 6 | 24 | **0.0001** |
| Control x Trehalose | 24 | 1 | 34 | 6 | 0.2350 |
| Control x Fucose | 24 | 1 | 17 | 13 | **0.0013** |
| Control x Fucoidan | 24 | 1 | 6 | 24 | **0.0001** |
| Trehalose x Fucose | 34 | 6 | 17 | 13 | **0.0137** |
| Trehalose x Fucoidan | 34 | 6 | 6 | 24 | **0.0001** |
| Fucose x Fucoidan | 17 | 13 | 6 | 24 | **0.0073** |

(D)

|  | Group 1 | | Group 2 | |  |
| --- | --- | --- | --- | --- | --- |
| Comparison | N Live | N Dead | N Live | N Dead | two-tailed *p* |
| PBS x Control | 15 | 1 | 19 | 3 | 0.6245 |
| PBS x Trehalose | 15 | 1 | 12 | 22 | **0.0001** |
| PBS x Fucose | 15 | 1 | 0 | 17 | **0.0001** |
| PBS x Fucoidan | 15 | 1 | 6 | 0 | 1.0000 |
| Control x Trehalose | 19 | 3 | 12 | 22 | **0.0003** |
| Control x Fucose | 19 | 3 | 0 | 17 | **0.0001** |
| Control x Fucoidan | 19 | 3 | 6 | 0 | 1.0000 |
| Trehalose x Fucose | 12 | 22 | 0 | 17 | **0.0044** |
| Trehalose x Fucoidan | 12 | 22 | 6 | 0 | **0.0048** |
| Fucose x Fucoidan | 0 | 17 | 6 | 0 | **0.0001** |
