# Supplementary material for: Digestive α-L-fucosidase activity in Rhodnius prolixus after blood feeding: effect of secretagogue and nutritional stimuli
Source: Front Physiol. 2023 Jul 19;14:1123414. doi: 10.3389/fphys.2023.1123414 (PMC10394381; doi:10.3389/fphys.2023.1123414)
Supplement: Supplementary file 15 [file Table6.docx]

Supplementary Table 6. Summary of the statistical analysis of data presented in Figure 2. (A) Comparisons of weights of insects before and after feeding with heparinated blood (Control), PBS, plasma and cell fractions. (B) Comparion of α-fucosidase activities of insects before and after feeding with heparinated blood, PBS, plasma and cell fractions, in the Anterior Midgut Contents (AMC), Anterior Midgut Tissue (AMT), Posterior Midgur Contents (PMC), and Posterior Midgut Tissue (PMT). BF – Before Feeding.

(A)

| Sample Subset | Type of test | Comparison | Results | |
| --- | --- | --- | --- | --- |
| Control Blood | Unpaired T test | Before x After feeding | t (175) = 25.78 | ***p* < 0.0001** |
| PBS | Unpaired T test | Before x After feeding | t (138) = 16.35 | ***p* < 0.0001** |
| Plasma | Unpaired T test | Before x After feeding | t (141) = 16.79 | ***p* < 0.0001** |
| Cell fraction | Unpaired T test | Before x After feeding | t (167) = 18.83 | ***p* < 0.0001** |
| Before feeding | One way ANOVA | Control x PBS x Plasma x Cell fraction | F (3, 372) = 1.157 | *p* = 0.3262 |
| After feeding | One way ANOVA | Control x PBS x Plasma x Cell fraction | F (3, 249) = 4.915 | ***p* = 0.0025** |
| After feeding | Tukey´s post hoc | Control x PBS | *p* = 0.4686 |  |
| After feeding | Tukey´s post hoc | Control x Plasma | ***p* = 0.0013** |  |
| After feeding | Tukey´s post hoc | Control x Cell Fraction | *p* = 0.8286 |  |
| After feeding | Tukey´s post hoc | PBS x Plasma | *p* = 0.1696 |  |
| After feeding | Tukey´s post hoc | PBS x Cell Fraction | *p* = 0.9068 |  |
| After feeding | Tukey´s post hoc | Plasma x Cell Fraction | ***p* = 0.0198** |  |

(B)

| Sample Subset | Type of test | Comparison | Results | |
| --- | --- | --- | --- | --- |
| AMC | Kruskal-Wallis | All groups | H (5, 28) = 19.9409 | ***p* = 0.00051** |
| AMC | Mann-Whitney U | BF x PBS | *p* = 0.0635 | M_1/2_ = 110.2, 214.5  U (5, 4) = 2 |
| AMC | Mann-Whitney U | BF x Control | ***p* = 0.0043** | M_1/2_ = 110.2, 1722  U (5, 6) = 0 |
| AMC | Mann-Whitney U | BF x Plasma | *p* = **0.0043** | M_1/2_ = 110.2, 716.6  U (5, 6) = 0 |
| AMC | Mann-Whitney U | BF x Cell Fraction | *p* = **0.0043** | M_1/2_ = 110.2, 1350  U (5, 6) = 0 |
| AMC | Mann-Whitney U | PBS x Control | ***p* = 0.0095** | M_1/2_ = 214.5, 1722  U (4, 6) = 0 |
| AMC | Mann-Whitney U | PBS x Plasma | *p* = 0.0667 | M_1/2_ = 214.5, 716.6  U (4, 6) = 0 |
| AMC | Mann-Whitney U | PBS x Cell Fraction | ***p* = 0.0095** | M_1/2_ = 214.5, 1350  U (4, 6) = 0 |
| AMC | Mann-Whitney U | Control x Plasma | *p* = 0.1320 | M_1/2_ = 1722, 716.6  U (6, 6) = 8 |
| AMC | Mann-Whitney U | Control x Cell Fraction | *p* = 0.3095 | M_1/2_ = 1722, 1350  U (6, 6) = 11 |
| AMC | Mann-Whitney U | Plasma x Cell Fraction | *p* = 0.3095 | M_1/2_ = 716.6, 1350  U (6, 6) = 11 |
| AMT | Kruskal-Wallis | All groups | H (5, 30) = 1.295 | *p* = 0.8623 |
| AMT | Mann-Whitney U | BF x PBS | *p* = 0.8182 | M_1/2_ = 190.3, 169.4  U (6, 6) = 16 |
| AMT | Mann-Whitney U | BF x Control | *p* = 0.4848 | M_1/2_ = 190.3, 201.4  U (6, 6) = 13 |
| AMT | Mann-Whitney U | BF x Plasma | *p* = 0.5887 | M_1/2_ = 190.3, 199.8  U (6, 6) = 14 |
| AMT | Mann-Whitney U | BF x Cell Fraction | *p* = 0.4848 | M_1/2_ = 190.3, 212.4  U (6, 6) = 13 |
| AMT | Mann-Whitney U | PBS x Control | *p* = 0.6991 | M_1/2_ = 169.4, 201.4  U (6, 6) = 15 |
| AMT | Mann-Whitney U | PBS x Plasma | *p* = 0.4848 | M_1/2_ = 169.4, 199.8  U (6, 6) = 13 |
| AMT | Mann-Whitney U | PBS x Cell Fraction | *p* = 0.5887 | M_1/2_ = 169.4, 212.4  U (6, 6) = 14 |
| AMT | Mann-Whitney U | Control x Plasma | *p* = 0.8182 | M_1/2_ = 201.4, 199.8  U (6, 6) = 16 |
| AMT | Mann-Whitney U | Control x Cell Fraction | *p* = 0.9372 | M_1/2_ = 201.4, 212.4  U (6, 6) = 17 |
| AMT | Mann-Whitney U | Plasma x Cell Fraction | *p* > 0.9999 | M_1/2_ = 199.8, 212.4  U (6, 6) = 18 |
| PMC | Kruskal-Wallis | All groups | H (5, 30) = 9.217 | *p* = 0.0559 |
| PMC | Mann-Whitney U | BF x PBS | *p* > 0.9999 | M_1/2_ = 266.5, 270.7  U (6, 6) = 18 |
| PMC | Mann-Whitney U | BF x Control | ***p* = 0.026** | M_1/2_ = 266.5, 505.3  U (6, 6) = 4 |
| PMC | Mann-Whitney U | BF x Plasma | *p* = 0.0931 | M_1/2_ = 266.5, 484.3  U (6, 6) = 7 |
| PMC | Mann-Whitney U | BF x Cell Fraction | *p* = 0.0931 | M_1/2_ = 266.5, 393.6  U (6, 6) = 7 |
| PMC | Mann-Whitney U | PBS x Control | *p* = 0.0649 | M_1/2_ = 270.7, 505.3  U (6, 6) = 6 |
| PMC | Mann-Whitney U | PBS x Plasma | *p* = 0.1320 | M_1/2_ = 270.7, 484.3  U (6, 6) = 8 |
| PMC | Mann-Whitney U | PBS x Cell Fraction | *p* = 0.0931 | M_1/2_ = 270.7, 393.6  U (6, 6) = 7 |
| PMC | Mann-Whitney U | Control x Plasma | *p* = 0.9372 | M_1/2_ = 505.3, 484.3  U (6, 6) = 17 |
| PMC | Mann-Whitney U | Control x Cell Fraction | *p* = 0.2403 | M_1/2_ = 505.3, 393.6  U (6, 6) = 10 |
| PMC | Mann-Whitney U | Plasma x Cell Fraction | *p* = 0.5887 | M_1/2_ = 484.3, 393.6  U (6, 6) = 14 |
| PMT | Kruskal-Wallis | All groups | H (5, 30) = 11.93 | ***p* = 0.0179** |
| PMT | Mann-Whitney U | BF x PBS | *p* = 0.6991 | M_1/2_ = 218.8, 231.6  U (6, 6) = 15 |
| PMT | Mann-Whitney U | BF x Control | *p* = 0.0931 | M_1/2_ = 218.8, 351.1  U (6, 6) = 7 |
| PMT | Mann-Whitney U | BF x Plasma | ***p* = 0.0022** | M_1/2_ = 218.8, 456.5  U (6, 6) = 0 |
| PMT | Mann-Whitney U | BF x Cell Fraction | ***p* = 0.0022** | M_1/2_ = 218.8, 359.9  U (6, 6) = 0 |
| PMT | Mann-Whitney U | PBS x Control | *p* = 0.1797 | M_1/2_ = 231.6, 351.1  U (6, 6) = 9 |
| PMT | Mann-Whitney U | PBS x Plasma | *p* = 0.0649 | M_1/2_ = 231.6, 456.5  U (6, 6) = 6 |
| PMT | Mann-Whitney U | PBS x Cell Fraction | *p* = 0.1320 | M_1/2_ = 231.6, 359.9  U (6, 6) = 8 |
| PMT | Mann-Whitney U | Control x Plasma | *p* = 0.6991 | M_1/2_ = 351.1, 456.5  U (6, 6) = 15 |
| PMT | Mann-Whitney U | Control x Cell Fraction | *p* = 0.6991 | M_1/2_ = 351.1, 359.9  U (6, 6) = 15 |
| PMT | Mann-Whitney U | Plasma x Cell Fraction | *p* = 0.8182 | M_1/2_ = 456.5, 359.9  U (6, 6) = 16 |
