# Supplementary material for: Digestive α-L-fucosidase activity in Rhodnius prolixus after blood feeding: effect of secretagogue and nutritional stimuli
Source: Front Physiol. 2023 Jul 19;14:1123414. doi: 10.3389/fphys.2023.1123414 (PMC10394381; doi:10.3389/fphys.2023.1123414)
Supplement: Supplementary file 17 [file Table10.docx]

Supplementary Table 10. Summary of the statistical analysis of data presented in Figure 4. (A) Comparisons of weights of insects before and after feeding with PBS, heparinated blood (Control), Human Hemoglobin (HH), Bovine Hemoglobin (BH), Bovine Albumin (BA), and Rabbit Albumin (RA). (B) Comparion of α-fucosidase activities of AMC samples prepared from insects before and after feeding with PBS, heparinated blood (Control), Human Hemoglobin (HH), Bovine Hemoglobin (BH), Bovine Albumin (BA), and Rabbit Albumin (RA). BF – Before Feeding. (C) Comparison of refusal rates in groups of insects that were offered the different diets above (Fisher´s exact test). (D) Comparion of mortality 5 days after feeding with the different diets above (Fisher´s exact test).

(A)

| Sample Subset | Type of test | Comparison | Results | |
| --- | --- | --- | --- | --- |
| PBS | Unpaired T test | Before x After feeding | t (27) = 12.59 | ***p* < 0.0001** |
| Control | Unpaired T test | Before x After feeding | t (22) = 11.49 | ***p* < 0.0001** |
| Human Hemoglobin | Unpaired T test | Before x After feeding | t (20) = 9.015 | ***p* < 0.0001** |
| Bovine Hemoglobin | Unpaired T test | Before x After feeding | t (23) = 16.92 | ***p* < 0.0001** |
| Bovine Albumin | Unpaired T test | Before x After feeding | t (22) = 11.79 | ***p* < 0.0001** |
| Rabbit Albumin | Unpaired T test | Before x After feeding | t (22) = 11.43 | ***p* < 0.0001** |
| Before feeding | One way ANOVA | PBS x Control x HH x BH x BA x RA | F (5, 57) = 2.889 | **P=0.0215** |
| Before feeding | Tukey´s post hoc | PBS x Control | *p* = 0.6272 | 95% C.I. = -0.006450 to 0.02095 |
| Before feeding | Tukey´s post hoc | PBS x HH | *p* = 0.5337 | 95% C.I. = -0.02113 to 0.005642 |
| Before feeding | Tukey´s post hoc | PBS x BH | *p* = 0.9982 | 95% C.I. = -0.01174 to 0.01566 |
| Before feeding | Tukey´s post hoc | PBS x BA | *p* = 0.7992 | 95% C.I. = -0.01915 to 0.007615 |
| Before feeding | Tukey´s post hoc | PBS x RA | *p* = 0.9870 | 95% C.I. = -0.01631 to 0.01046 |
| Before feeding | Tukey´s post hoc | Control x HH | ***p* = 0.0196** | 95% C.I. = -0.02838 to -0.001608 |
| Before feeding | Tukey´s post hoc | Control x BH | *p* = 0.8631 | 95% C.I. = -0.01899 to 0.008410 |
| Before feeding | Tukey´s post hoc | Control x BA | *p* = 0.0610 | 95% C.I. = -0.02640 to 0.0003646 |
| Before feeding | Tukey´s post hoc | Control x RA | *p* = 0.2354 | 95% C.I. = -0.02356 to 0.003210 |
| Before feeding | Tukey´s post hoc | HH x BH | *p* = 0.2832 | 95% C.I. = -0.003682 to 0.02309 |
| Before feeding | Tukey´s post hoc | HH x BA | *p* = 0.5355 | 95% C.I. = -0.02111 to 0.005655 |
| Before feeding | Tukey´s post hoc | HH x RA | *p* = 0.8888 | 95% C.I. = -0.01827 to 0.008500 |
| Before feeding | Tukey´s post hoc | BH x BA | *p* = 0.9872 | 95% C.I. = -0.01022 to 0.01591 |
| Before feeding | Tukey´s post hoc | BH x RA | *p* = | 95% C.I. = |
| Before feeding | Tukey´s post hoc | BA x RA | *p* = | 95% C.I. = |
| After feeding | One way ANOVA | PBS x Control x HH x BH x BA x RA | F (5, 79) = 2.001 | P=0.0876 |
| After feeding | Tukey´s post hoc | PBS x Control | *p* = 0.0412 | 95% C.I. = 0.0006073 to 0.04901 |
| After feeding | Tukey´s post hoc | PBS x HH | *p* = 0.9663 | 95% C.I. = -0.01888 to 0.03318 |
| After feeding | Tukey´s post hoc | PBS x BH | *p* = 0.9970 | 95% C.I. = -0.01990 to 0.02756 |
| After feeding | Tukey´s post hoc | PBS x BA | *p* = 0.9696 | 95% C.I. = -0.01810 to 0.03136 |
| After feeding | Tukey´s post hoc | PBS x RA | *p* = 0.9882 | 95% C.I. = -0.01937 to 0.03009 |
| After feeding | Tukey´s post hoc | Control x HH | *p* = 0.4325 | 95% C.I. = -0.04534 to 0.01003 |
| After feeding | Tukey´s post hoc | Control x BH | *p* = 0.1690 | 95% C.I. = -0.04651 to 0.004557 |
| After feeding | Tukey´s post hoc | Control x BA | *p* = 0.3483 | 95% C.I. = -0.04464 to 0.008289 |
| After feeding | Tukey´s post hoc | Control x RA | *p* = 0.2746 | 95% C.I. = -0.04592 to 0.007012 |
| After feeding | Tukey´s post hoc | HH x BH | *p* = 0.9992 | 95% C.I. = -0.03059 to 0.02395 |
| After feeding | Tukey´s post hoc | HH x BA | *p* > 0.9999 | 95% C.I. = -0.02867 to 0.02763 |
| After feeding | Tukey´s post hoc | HH x RA | *p* > 0.9999 | 95% C.I. = -0.02994 to 0.02635 |
| After feeding | Tukey´s post hoc | BH x BA | *p* = 0.9996 | 95% C.I. = -0.02323 to 0.02884 |
| After feeding | Tukey´s post hoc | BH x RA | *p* > 0.9999 | -0.02451 to 0.02756 |
| After feeding | Tukey´s post hoc | BA x RA | *p* > 0.9999 | -0.02823 to 0.02567 |

(B)

| Sample Subset | Type of test | Comparison | Results | |
| --- | --- | --- | --- | --- |
| AMC | ANOVA | All groups | F (6, 92) = 6.314 | ***p* < 0.0001** |
| AMC | Tukey´s post hoc | BF x PBS | *p* = 0.9997 | 95% C.I. = -1617 to 1236 |
| AMC | Tukey´s post hoc | BF x Control | ***p* = 0.0019** | 95% C.I. = -3112 to -460.9 |
| AMC | Tukey´s post hoc | BF x HH | *p* = 0.7984 | 95% C.I. = -2233 to 813.9 |
| AMC | Tukey´s post hoc | BF x BH | ***p* = 0.0002** | 95% C.I. = -3494 to -774.7 |
| AMC | Tukey´s post hoc | BF x RA | ***p* = 0.0379** | 95% C.I. = -3098 to -51.42 |
| AMC | Tukey´s post hoc | BF x BA | *p* = 0.5029 | 95% C.I. = -2410 to 562.6 |
| AMC | Tukey´s post hoc | PBS x Control | ***p* = 0.0102** | 95% C.I. = -2948 to -244.3 |
| AMC | Tukey´s post hoc | PBS x HH | *p* = 0.9500 | 95% C.I. = -2065 to 1027 |
| AMC | Tukey´s post hoc | PBS x BH | ***p* = 0.0010** | 95% C.I. = -3329 to -558.8 |
| AMC | Tukey´s post hoc | PBS x RA | *p* = 0.1101 | 95% C.I. = -2931 to 161.8 |
| AMC | Tukey´s post hoc | PBS x BA | *p* = 0.7648 | 95% C.I. = -2243 to 776.3 |
| AMC | Tukey´s post hoc | Control x HH | *p* = 0.2886 | 95% C.I. = -377.1 to 2531 |
| AMC | Tukey´s post hoc | Control x BH | *p* = 0.9825 | 95% C.I. = -1629 to 933.5 |
| AMC | Tukey´s post hoc | Control x RA | *p* = 0.9994 | 95% C.I. = -1242 to 1666 |
| AMC | Tukey´s post hoc | Control x BA | *p* = 0.5266 | 95% C.I. = -552.5 to 2278 |
| AMC | Tukey´s post hoc | HH x BH | *p* = 0.0688 | 95% C.I. = -2910 to 60.35 |
| AMC | Tukey´s post hoc | HH x RA | *p* = 0.6864 | 95% C.I. = -2502 to 771.1 |
| AMC | Tukey´s post hoc | HH x BA | *p* = 0.9996 | 95% C.I. = -1816 to 1388 |
| AMC | Tukey´s post hoc | BH x RA | *p* = 0.9154 | 95% C.I. = -925.7 to 2044 |
| AMC | Tukey´s post hoc | BH x BA | *p* = 0.1638 | 95% C.I. = -236.6 to 2657 |
| AMC | Tukey´s post hoc | RA x BA | *p* = 0.8826 | 95% C.I. = -950.9 to 2253 |

(C)

|  | Group 1 | | Group 2 | |  |
| --- | --- | --- | --- | --- | --- |
| Comparison | N Fed | N Refused | N Fed | N Refused | two-tailed *p* |
| PBS x Control | 26 | 3 | 24 | 1 | 0.6149 |
| PBS x HH | 26 | 3 | 17 | 11 | **0.0148** |
| PBS x BH | 26 | 3 | 21 | 7 | 0.1789 |
| PBS x RA | 26 | 3 | 23 | 6 | 0.4703 |
| PBS x BA | 26 | 3 | 18 | 10 | **0.0295** |
| Control x HH | 24 | 1 | 17 | 11 | **0.0026** |
| Control x BH | 24 | 1 | 21 | 7 | 0.0533 |
| Control x RA | 24 | 1 | 23 | 6 | 0.1076 |
| Control x BA | 24 | 1 | 18 | 10 | **0.0058** |
| HH x BH | 17 | 11 | 21 | 7 | 0.3911 |
| HH x BA | 17 | 11 | 18 | 10 | 1.0000 |
| HH x RA | 17 | 11 | 23 | 6 | 0.1550 |
| BH x RA | 21 | 7 | 23 | 6 | 0.7598 |
| BH x BA | 21 | 7 | 18 | 10 | 0.5619 |
| RA x BA | 23 | 6 | 18 | 10 | 0.2483 |

(D)

|  | Group 1 | | Group 2 | |  |
| --- | --- | --- | --- | --- | --- |
| Comparison | N Live | N Dead | N Live | N Dead | two-tailed *p* |
| PBS x Control | 15 | 1 | 19 | 3 | 0.6245 |
| PBS x HH | 15 | 1 | 13 | 5 | 0.1801 |
| PBS x BH | 15 | 1 | 20 | 2 | 1.0000 |
| PBS x RA | 15 | 1 | 17 | 7 | 0.1140 |
| PBS x BA | 15 | 1 | 16 | 2 | 1.0000 |
| Control x HH | 19 | 3 | 13 | 5 | 0.4295 |
| Control x BH | 19 | 3 | 20 | 2 | 1.0000 |
| Control x RA | 19 | 3 | 17 | 7 | 0.2894 |
| Control x BA | 19 | 3 | 16 | 2 | 1.0000 |
| HH x BH | 13 | 5 | 20 | 2 | 0.2110 |
| HH x BA | 13 | 5 | 16 | 2 | 0.4018 |
| HH x RA | 13 | 5 | 17 | 7 | 1.0000 |
| BH x RA | 20 | 2 | 17 | 7 | 0.1386 |
| BH x BA | 20 | 2 | 16 | 2 | 1.0000 |
| RA x BA | 17 | 7 | 16 | 2 | 0.2578 |
